# Macrophage migration inhibitory factor in atrial fibrillation

**DOI:** 10.3389/fimmu.2026.1874330

**Published:** 2026-07-02

**Authors:** Xize Wu, Shan Gao, Ruiying Wang, Qicheng Cai, Minglin Ruan, Jiaqi Ren, Yuxi Huang, Yue Li, Lihong Gong

**Affiliations:** 1 Liaoning University of Traditional Chinese Medicine, Shenyang, China; 2 Affiliated Hospital of Liaoning University of Traditional Chinese Medicine, Shenyang, China

**Keywords:** atrial fibrillation, electrical remodeling, macrophage migration inhibitory factor, oxidative stress, structural remodeling

## Abstract

Macrophage migration inhibitory factor (MIF) is a multifunctional upstream cytokine that has attracted increasing attention for its role in the initiation and perpetuation of atrial fibrillation (AF). This review systematically discusses the dual regulatory roles of MIF in AF and its potential as both a biomarker and a therapeutic target. Mechanistically, MIF drives atrial electrical remodeling by promoting the release of pro-inflammatory cytokines, modulating ion channels, disrupting calcium homeostasis, and downregulating connexin 43. Concurrently, MIF promotes atrial structural remodeling and fibrosis through the activation of fibroblasts, enhancement of collagen deposition, and modulation of the TGF-β/Smad signaling pathway. Clinical studies have demonstrated that circulating MIF levels are independently associated with AF type, disease burden, the extent of atrial fibrosis, and long-term adverse outcomes, including heart failure, stroke, and myocardial infarction. MIF possesses an N-terminal tautomerase activity and a thiol-protein oxidoreductase (TPOR) activity mediated by its Cys57-Ala-Leu-Cys60 (CALC) motif, the latter serving as the structural basis for its antioxidant functions. Reflecting this property, the dynamic perioperative changes in MIF exhibit a biphasic predictive value for postoperative AF (POAF). Therapeutically, direct MIF inhibition (e.g., with 4-IPP) or blockade of downstream signaling (e.g., with CXCR2 antagonists) has shown antiarrhythmic potential in animal models; however, non-selective pan-inhibition may inadvertently ablate the endogenous antioxidant and cardioprotective signals of MIF. Future research should focus on elucidating the molecular switch that governs the functional transition of MIF, developing highly selective drugs targeting the disease-related conformational isoform oxMIF to precisely block pathogenic signaling, validating the existence of a MIF-TGF-β positive feedback loop in atrial fibroblasts, implementing time-window-based intervention strategies, and incorporating MIF promoter polymorphisms into personalized patient stratification. Addressing these priorities will be essential to advance the clinical translation of MIF-targeted therapies for atrial fibrillation.

## 1 Introduction

Atrial fibrillation (AF) is the most common cardiac arrhythmia encountered in clinical practice. Owing to global population aging and the rising prevalence of cardiovascular and metabolic risk factors, including hypertension, diabetes, and obesity, the global burden of AF has increased rapidly over recent decades. According to the 2021 Global Burden of Disease Study, approximately 52.55 million individuals were affected by AF and atrial flutter worldwide in 2021, representing a 137% increase from 1990 (1). Furthermore, the Framingham Heart Study indicates that the lifetime risk of developing AF exceeds 25% among individuals aged 40 years and older (2). Beyond its detrimental effects on quality of life, AF is an independent risk factor for ischemic stroke, heart failure, and all-cause mortality, imposing a substantial burden on healthcare systems. Although catheter ablation reduces AF burden, recurrence rates remain considerable. Similarly, while anticoagulation and left atrial appendage occlusion lower stroke risk, they do not modify the underlying atrial remodeling processes that drive AF initiation and persistence (3). Therefore, elucidating the molecular mechanisms governing AF pathophysiology and identifying novel therapeutic targets are urgent priorities for translation.

The pathophysiology of AF involves complex, multi-level interactions encompassing electrical, structural, and autonomic remodeling. Electrical remodeling is predominantly characterized by shortening of action potential duration and reduction of the effective refractory period. Concurrently, structural remodeling manifests as atrial dilation, cardiomyocyte hypertrophy, and progressive interstitial fibrosis. Emerging evidence indicates that immune-mediated mechanisms—particularly inflammation-driven structural remodeling—play a critical pathophysiological role in AF initiation and maintenance (4). Inflammatory responses enhance AF susceptibility through at least two interconnected pathways: altering electrophysiological properties via modulation of ion channel function and promoting structural remodeling through extracellular matrix deposition and fibroblast activation (5). Given these observations, anti-inflammatory and immunomodulatory strategies have attracted growing interest for AF management. However, current research remains largely focused on downstream cytokine networks and canonical signaling pathways. The upstream, multifunctional cytokine macrophage migration inhibitory factor (MIF), though well-recognized in other cardiovascular diseases, remains relatively underexplored in the specific context of atrial remodeling in AF.

MIF is a highly conserved, multifunctional cytokine that occupies an upstream position in immune activation cascades. It is constitutively preformed and rapidly released in response to hypoxia, infection, or glucocorticoids, thereby functioning as a first-response mediator that initiates and amplifies inflammatory cascades (6). The pathophysiological significance of MIF in cardiovascular diseases—including atherosclerosis, myocardial ischemia-reperfusion injury, and heart failure—is well established. Research on its role in AF is now expanding, with emerging evidence indicating that MIF contributes to AF pathophysiology through multiple interconnected mechanisms. Clinical studies have reported significantly elevated serum MIF levels in AF patients (7, 8). Mechanistically, MIF derived from atrial fibroblasts promotes the recruitment and activation of atrial macrophages in postoperative AF (POAF) (9). In obstructive sleep apnea-induced AF, HIF1α upregulates MIF expression in cardiomyocytes; secreted MIF then activates CD74-dependent NF-κB signaling in macrophages, driving M1 polarization and amplifying inflammatory responses (10). However, AF is a heterogeneous syndrome encompassing diverse clinical subtypes, including POAF, valvular AF, obesity-related AF, and persistent AF. The relative contribution of MIF to AF pathophysiology may differ across these subtypes, given the varying degrees of inflammation, oxidative stress, and fibrotic remodeling that characterize each condition. Despite these advances, current understanding of MIF in AF remains fragmented, and a systematic synthesis of its multifaceted roles is lacking. This review aims to provide guidance for future research and clinical practice by comprehensively integrating existing evidence on MIF’s involvement in AF initiation and maintenance, including cellular and molecular mechanisms, key biological processes, clinical correlations and prognostic implications, and therapeutic targeting strategies.

## 2 Biological properties of MIF

### 2.1 Cellular origins, tissue distribution, and molecular characteristics

MIF was initially identified as a factor secreted by activated T lymphocytes that inhibits macrophage migration (11, 12). Subsequent studies have shown that MIF is produced by multiple immune cells, including dendritic cells, B cells, T cells, macrophages, monocytes, and neutrophils (13, 14). Under stress conditions, it is also synthesized and released by endothelial cells, epithelial cells, cardiomyocytes, and endocrine cells (15–17). MIF is widely expressed across various tissues, such as the immune, cardiovascular, endocrine, and central nervous systems, and can be secreted into the circulation and local body fluids, making it a potential disease biomarker (18).

The human MIF gene is located on chromosome 22q11.2 and contains three exons and two introns, encoding a 115-amino acid polypeptide (19, 20). MIF forms a homotrimer, with each monomer consisting of two antiparallel α-helices and a four-stranded β-sheet (21). Promoter polymorphisms of MIF, particularly MIF-173G/C (rs755622) and the MIF-794 CATT5–8 microsatellite (rs5844572), have been linked to susceptibility to several cardiovascular diseases. A meta-analysis of 8,488 participants demonstrated that the MIF rs755622 G/C polymorphism is significantly associated with coronary artery disease, with the C allele conferring increased coronary artery disease susceptibility (22). This association has been further validated in acute coronary syndrome, where the rs755622 C allele was associated with higher acute coronary syndrome risk (Adjusted Odds Ratio = 1.278, 95% CI: 1.042–1.567) and elevated plasma MIF levels (23). Moreover, the GG genotype of rs755622 has been implicated in heart failure, increasing heart failure risk by approximately 4.25-fold compared with controls (24). Regarding the CATT5–8 microsatellite, the (CATT)7 allele has been associated with increased severity of carotid artery atherosclerosis in ischemic stroke patients (25), and the 6/7 genotype of the MIF -794 (CATT)5–8 polymorphism has been linked to susceptibility to acute coronary syndrome in a western Mexican population (26). Despite these well-documented associations in other cardiovascular conditions, the role of these functional promoter polymorphisms in atrial fibrillation remains completely unexplored. Given that MIF expression levels correlate with AF burden and atrial remodeling, investigating whether these polymorphisms are associated with AF susceptibility, disease progression, or response to MIF-targeted therapies represents an important direction for future research.

### 2.2 The MIF receptor system and downstream signaling network

MIF’s biological functions depend on its interaction with cell surface receptors. CD74 is the primary high-affinity receptor, but its short intracellular domain lacks signaling motifs and therefore requires co-receptor assistance (27–29). CD44 serves as a key co-receptor: upon MIF binding to CD74, CD44 is recruited to form a functional signaling complex that activates Src family kinases (28). In addition, MIF acts as an alternative ligand for the CXC chemokine receptors CXCR2, CXCR4, and CXCR7. Functional signaling via CXCR4 requires the formation of a CD74/CXCR4 heteromeric complex, which mediates MIF-specific pathways such as AKT activation in monocytes and may contribute to leukocyte chemotaxis, migration, and angiogenesis (30, 31). Similarly, CXCR2 forms a complex with CD74 to promote monocyte adhesion, whereas CXCR7 complexes with both CXCR4 and CD74 for B-cell chemotaxis (30, 31). This ability to interact with multiple receptors and form heteromeric complexes enables MIF to flexibly activate diverse signaling pathways depending on the cellular microenvironment.

Through these receptor systems, MIF activates key downstream pathways, including NF-κB, ERK1/2, PI3K/AKT, and AMPK6, (6, 32, 33). MIF can also enter cells via endocytosis and directly interact with intracellular proteins such as JAB1/CSN5, p53, and thioredoxin-interacting protein (TXNIP), thereby regulating cellular functions (32). The parallel and synergistic actions of these two signaling modes, together with the involvement of the MIF homolog D-DT (MIF-2), form the complex basis of the MIF signaling network. These pathways regulate cell proliferation, apoptosis, cytokine production, immune responses, and cell adhesion, providing the molecular basis for MIF’s involvement in numerous pathological conditions, including cancer, autoimmune diseases, infectious diseases, and cardiovascular disease.

MIF exerts a wide range of biological functions that have been extensively reviewed elsewhere (6). Briefly, MIF regulates macrophage polarization, T/B cell differentiation, and pro-inflammatory cytokine expression, acting as an inflammatory amplifier in chronic diseases. It also serves as a glucocorticoid counter-regulator, antagonizing steroid immunosuppression to fine-tune the inflammatory balance (34). In the cardiovascular system, the roles of MIF in myocardial ischemia/reperfusion (I/R) injury (35), ischemic heart disease (36), and inflammatory cardiomyopathy (37) have been comprehensively reviewed. MIF exhibits dual effects: it protects the heart by activating AMPK signaling, reducing oxidative stress and apoptosis, and enhancing myocardial energy metabolism, while its pro-inflammatory properties may also exacerbate disease progression. Comprehensive discussions of MIF in cancer (38), metabolism (39, 40), neurodegeneration (41), and infection (42) can be found in the cited reviews; here we focus on the redox-dependent structural features directly relevant to AF pathophysiology.

### 2.3 Redox-dependent structural features of MIF

Oxidative stress is a major driver of atrial remodeling in AF. MIF possesses both direct and indirect antioxidant activities that are mediated by distinct structural domains, and understanding these redox-dependent features may reveal novel therapeutic opportunities for AF. MIF is a unique cytokine that possesses two distinct evolutionarily conserved enzyme activities: an N-terminal tautomerase activity (mediated by Pro1) and a thiol-protein oxidoreductase (TPOR) activity (mediated by its Cys57-Ala-Leu-Cys60 motif). While the physiological substrates of the tautomerase activity remain elusive, both enzymatic functions contribute to the complex roles of MIF in inflammation and redox regulation (43).

The antioxidant actions of MIF are mediated by direct free radical scavenging and indirect activation of antioxidant defense systems, involving multiple structural domains. The direct antioxidant activity of MIF, also known as its TPOR activity, depends primarily on the Cys57-Ala-Leu-Cys60 (CALC) motif (44). This motif forms a canonical Cys-Xaa-Xaa-Cys (CXXC) redox-active center. The side-chain thiol groups (–SH) of Cys and Cys can reversibly form or break intramolecular disulfide bonds, directly reducing oxidized proteins or scavenging reactive oxygen species. Site-directed mutagenesis studies have confirmed that disruption of this motif (e.g., Cys60Ser mutation) completely abolishes MIF’s TPOR activity, demonstrating that the CALC motif is essential for its direct antioxidant function (45).

The antioxidant activity of MIF is regulated by Cys81. Although Cys81 does not directly participate in catalysis, its thiol group undergoes post-translational modifications (e.g., S-nitrosylation or oxidation) that act as a molecular switch. Under oxidative stress conditions such as I/R, the redox state of Cys81 triggers a conformational transition from reduced MIF (redMIF) to oxidized MIF (oxMIF) (46, 47). This redox-dependent conformational change modulates the TPOR activity of the CALC motif and influences MIF’s cardioprotective effects (47). Thus, Cys81 serves as a fine-tuning regulator that may underlie the switch between different MIF functions under distinct physiological or pathological conditions.

In addition to its direct enzymatic activity, MIF indirectly activates the endogenous antioxidant defense system—the Nrf2-ARE pathway—through its N-terminal proline (Pro1)-mediated tautomerase activity (48–50). Small molecules such as BTZO-1, which specifically bind to the Pro1 site, enhance MIF’s ability to transactivate the expression of phase II detoxifying enzymes and antioxidant proteins via the ARE element (50). This indirect mechanism enables MIF to provide more sustained protection by boosting the cell’s own antioxidant capacity, and its functional integrity also depends on the presence of Pro1.

## 3 MIF-driven mechanisms of AF

### 3.1 MIF and inflammation

**Figure 1:**
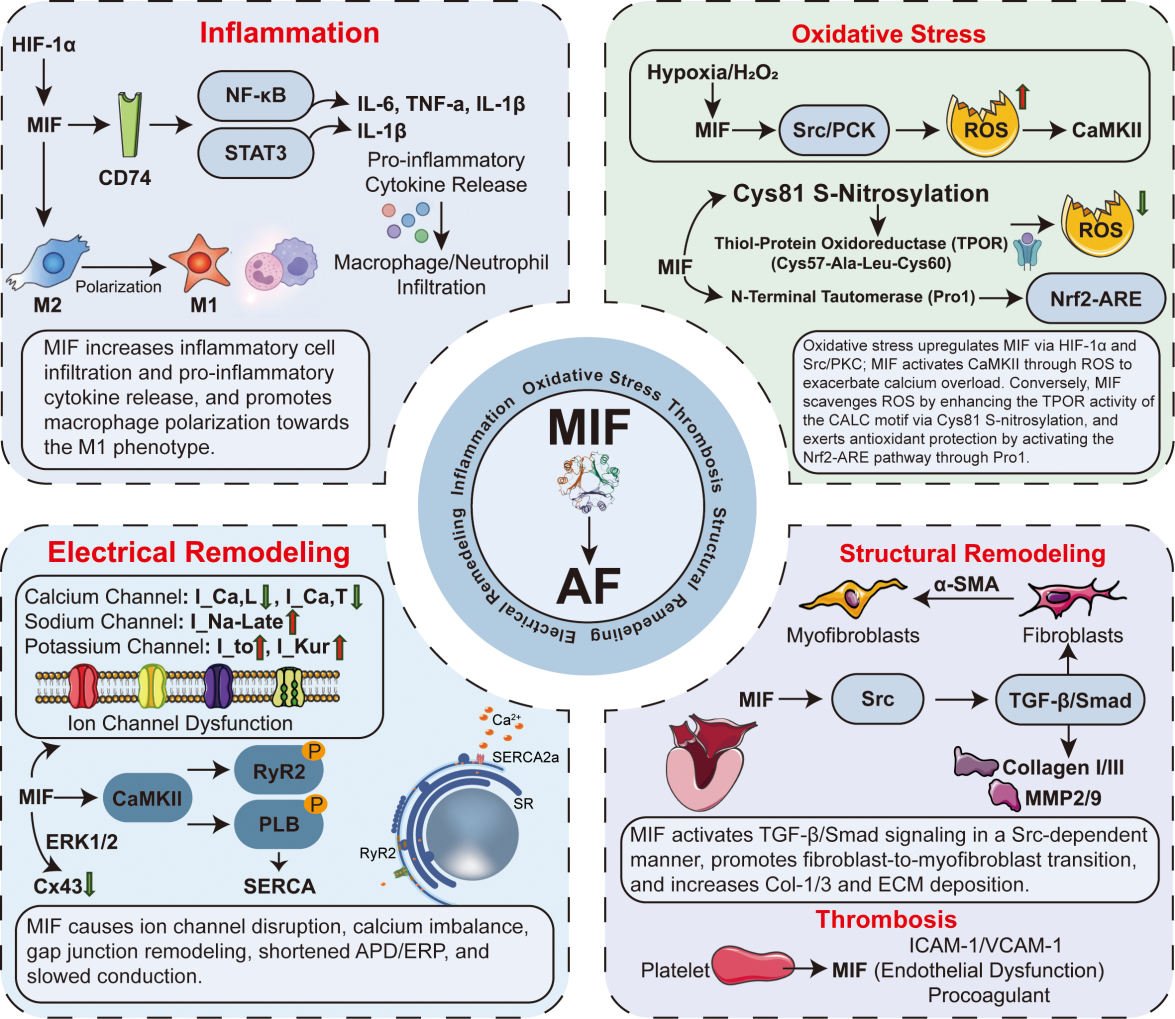
Schematic illustration of the dual roles of MIF in AF. MIF drives inflammatory responses and promotes macrophage polarization toward the M1 phenotype; promotes ROS production via the Src/PKC pathway; induces electrical remodeling (suppression of L-type and T-type calcium currents, enhancement of late sodium current and repolarizing potassium currents, downregulation of connexin 43, CaMKII-mediated Ca^2+^ dysregulation through RyR2/NCX/PLB) and structural remodeling (fibroblast-to-myofibroblast transition, collagen deposition, activation of the TGF-β/Smad axis). Ultimately, MIF increases susceptibility to AF, promotes AF persistence, and contributes to the development of POAF. In contrast, under acute ischemia/reperfusion conditions, MIF directly scavenges reactive oxygen species via S-nitrosylation of Cys81, which enhances the thiol-protein oxidoreductase (TPOR) activity of the CALC motif; it also indirectly exerts antioxidant effects by activating the Nrf2-ARE pathway through its Pro1 tautomerase activity. These cardioprotective actions reduce early-phase POAF. Thus, MIF exhibits context-dependent dual effects in AF: pathogenic under chronic/subacute conditions and protective under acute ischemia/reperfusion.

**Figure 2:**
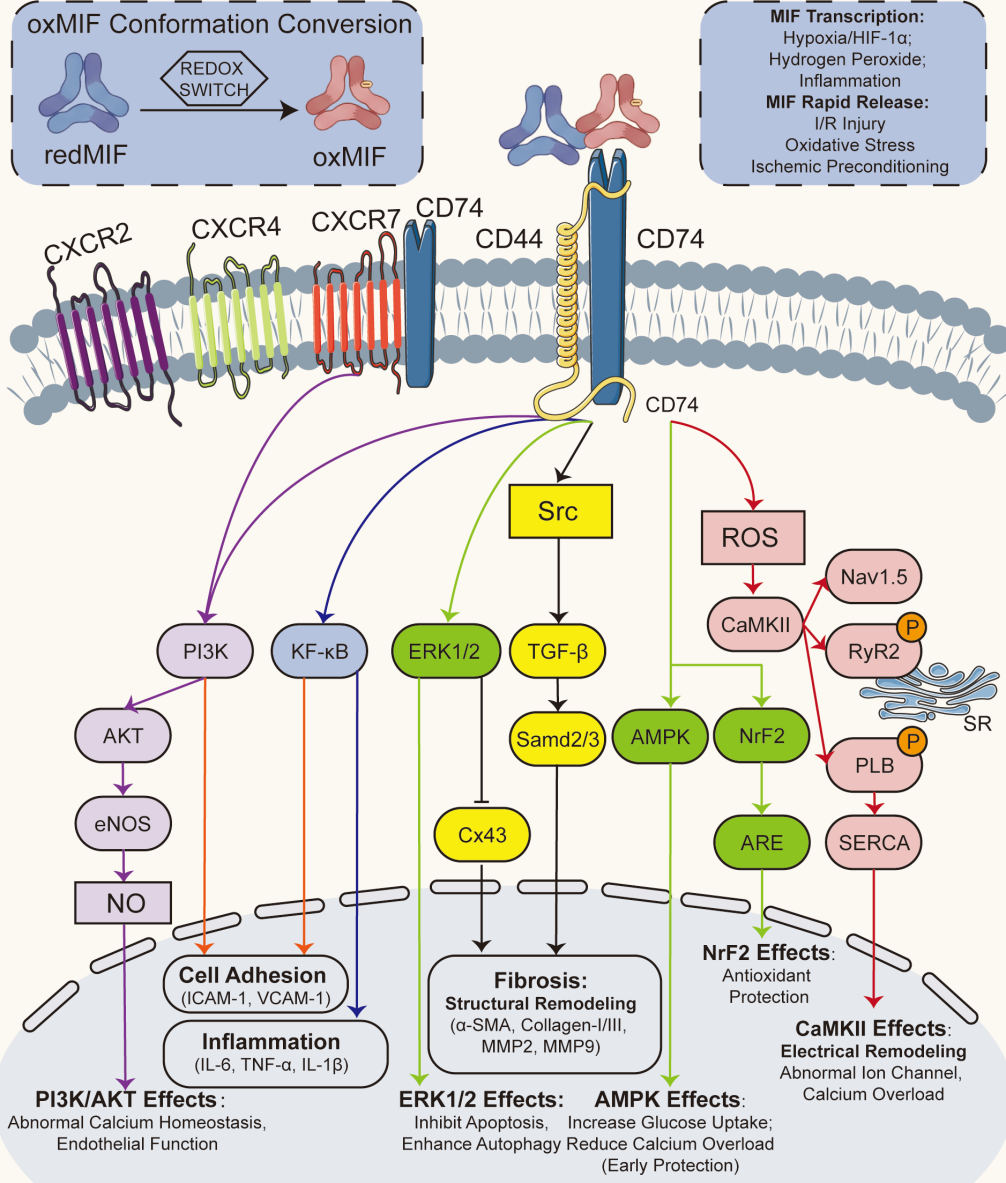
Signal cascade of MIF in AF. Under chronic or subacute pathological conditions (e.g., hypoxia, inflammation), MIF is transcriptionally upregulated in atrial fibroblasts and macrophages. In contrast, under acute ischemia/reperfusion or oxidative stress conditions, cardiomyocytes rapidly release pre-stored MIF. MIF drives the progression of atrial fibrillation (AF) by activating downstream signaling pathways including PI3K/AKT, NF-κB, ERK1/2, Src, TGF-β/Smad, and CaMKII through CD74/CD44, CXCR2, CXCR4, and CXCR7 receptors. Meanwhile, MIF exerts cardioprotective effects by promoting glucose uptake via the CD74/AMPK pathway, activating NRF2/ARE through its Pro1 tautomerase activity to exert antioxidant effects, and inhibiting apoptosis while enhancing autophagy via ERK1/2.

The pleiotropic roles of MIF in AF pathogenesis and protection are summarized in **Figure 1**. The signal cascade of MIF in AF is summarized in **Figure 2**. Chronic low-grade inflammation is a key upstream event in the development and maintenance of AF. A large-scale population study of 5,806 participants confirmed that systemic inflammation levels are independently associated with AF prevalence and future risk (51). Elevated circulating levels of inflammatory markers such as C-reactive protein, TNF-α, IL-1β, and IL-6 are associated with AF progression (52, 53). Moreover, AF itself can further exacerbate the inflammatory response, creating a vicious cycle in which AF begets AF (54).

Among the upstream regulators of this inflammatory cascade, MIF plays a central role by activating the NF-κB signaling pathway. The primary molecular basis for MIF’s pro-inflammatory function is its binding to the CD74 receptor, which triggers NF-κB activation and promotes the release of pro-inflammatory cytokines such as IL-6 and TNF-α (6). This pro-inflammatory axis has been validated in AF models. In a chronic intermittent hypoxia-induced AF model (simulating obstructive sleep apnea), HIF-1α upregulates MIF transcription in atrial myocytes. The increased MIF then binds to CD74 on the macrophage surface, activating NF-κB and promoting macrophage polarization toward the pro-inflammatory M1 phenotype, which in turn releases TNF-α, IL-1β, and IL-6, thereby exacerbating atrial remodeling and AF susceptibility (10). In a mouse model of POAF, the MIF-specific inhibitor 4-iodo-6-phenylpyrimidine (4-IPP) significantly reduces atrial macrophage infiltration, lowers the incidence of POAF, and shortens its duration. These findings directly confirm that MIF drives inflammation by recruiting macrophages, thereby triggering AF (9).

### 3.2 MIF and oxidative stress

Oxidative stress, defined as an imbalance between reactive oxygen species (ROS) production and endogenous antioxidant capacity, promotes atrial remodeling and AF maintenance by modulating calcium-handling proteins and ion channels (55). MIF and oxidative stress have a complex bidirectional relationship: oxidative stress triggers MIF expression and release, while MIF exerts antioxidant protection through its intrinsic TPOR activity (46). However, under certain pro-oxidant conditions, MIF can also contribute to ROS-mediated arrhythmogenesis.

#### 3.2.1 Oxidative stress triggers MIF expression and arrhythmogenic effects

In HL-1 mouse atrial myocytes, hydrogen peroxide (H_2_O_2_) stimulates a dose-dependent upregulation of MIF protein and mRNA expression. This effect is completely blocked by catalase and depends on Src and PKC signaling pathways (56). In the obstructive sleep apnea-induced AF model described above (section 3.1), chronic intermittent hypoxia promotes MIF transcription and expression in atrial myocytes via HIF-1α, highlighting the key role of oxidative/hypoxic stress as an upstream inducer of MIF (10). Furthermore, in myocardial I/R injury, cardiomyocytes secrete MIF under oxidative stress conditions, and its release depends on the PKC pathway rather than ERK1/2 (57).

Under these pro-oxidant conditions, MIF can paradoxically enhance ROS production. MIF increases ROS generation, which in turn enhances late sodium currents through the ROS/CaMKII signaling pathway in pulmonary vein cardiomyocytes, leading to intracellular Na^+^ overload. The resulting Na^+^ overload then exacerbates Ca^2+^ overload via reverse-mode Na^+^/Ca^2+^ exchange, thereby inducing delayed afterdepolarizations and triggered activity (58). This pro-oxidant role is further supported by the finding that exogenous MIF directly triggers ROS generation (59).

#### 3.2.2 Antioxidant protective functions of MIF

MIF also possesses intrinsic antioxidant functions, conferring a dual role in oxidative stress regulation (35). In cardiac surgery patients, those undergoing coronary artery bypass grafting with cardiopulmonary bypass exhibit significantly elevated postoperative MIF levels. Their endogenous peroxidase activity and thioredoxin levels are also significantly higher than in patients undergoing off-pump surgery. Furthermore, perioperative MIF release is significantly associated with a reduced incidence of POAF, suggesting that MIF may exert a protective effect against POAF by enhancing antioxidant capacity (60).

Mechanistically, Cys81 acts as a redox-sensitive switch that enhances MIF’s TPOR activity (mediated by the CALC motif) upon S-nitrosylation, directly scavenging ROS and reducing cardiomyocyte apoptosis (46, 61). In MIF-deficient cardiac fibroblasts, ROS levels increase 2.3-fold under oxidative stress, and re-expression of MIF reverses this effect (62). In a mouse model of myocardial I/R injury, MIF-deficient hearts show a reduced glutathione/oxidized glutathione (GSH/GSSG) ratio, increased protein oxidation, greater mitochondrial damage, larger infarct size, and failure to recover left ventricular systolic function (62). In a stress-induced cardiac hypertrophy model, MIF deficiency leads to a 10-fold increase in NADPH oxidase mRNA and a 4-fold increase in fibrosis (63). Collectively, these findings indicate that MIF exerts cardioprotective effects by maintaining redox homeostasis, with its net effect depending on the pathological stage and local microenvironment.

### 3.3 MIF-mediated atrial remodeling

#### 3.3.1 Atrial electrical remodeling

Electrical remodeling in AF is characterized by shortened action potential duration and effective refractory period, slowed conduction velocity, and increased spatial heterogeneity. MIF contributes to this process through three interconnected mechanisms: ion channel dysfunction, calcium homeostasis dysregulation, and gap junction remodeling.

##### 3.3.1.1 Ion channel dysfunction and calcium handling abnormalities

MIF differentially regulates L-type calcium channels depending on cell type. In atrial myocytes, MIF suppresses the L-type calcium channel current (I_Ca,L) and impairs its recovery from inactivation (64). In contrast, one study in rabbit pulmonary vein cardiomyocytes reported that MIF, acting through the ROS/CaMKII pathway, contributes to an increase in I_Ca,L as part of a broader pro-arrhythmic cascade (58). In atrial tissue from AF patients or in MIF-treated HL-1 cardiomyocytes, the density of I_Ca,L and the expression of its α1C subunit are significantly reduced (64), which shortens action potential duration and the effective refractory period. MIF also inhibits the T-type calcium channel current (I_Ca,T) and downregulates its α1G and α1H subunits via c-Src kinase activation; Src inhibitors (genistein, PP1) reverse this suppression (65).

As described in section 3.2.1, MIF increases the late sodium current (I_Na-Late) in pulmonary vein cardiomyocytes, leading to intracellular Na^+^ overload and, via reverse-mode Na^+^/Ca^2+^ exchange, Ca^2+^ overload that triggers delayed afterdepolarizations (58). MIF also elevates ROS levels, further enhancing I_Na-Late through the ROS-CaMKII pathway; pharmacological inhibition of CaMKII (KN93), I_Na-Late (ranolazine), or ROS (N-mercaptopropionyl glycine) abolishes these pro-arrhythmic effects (58). Additionally, MIF enhances the repolarizing potassium currents—transient outward potassium current (I_to) and ultra-rapid delayed rectifier potassium current (I_Kur)—through CD74 signaling, further shortening action potential duration and promoting re-entry; neutralizing anti-CD74 antibodies attenuate MIF-induced electrical dysregulation (66).

Beyond direct ion channel modulation, MIF disrupts intracellular calcium homeostasis. In HL-1 atrial myocytes and primary cardiomyocytes, MIF activates CaMKII, which phosphorylates RyR2 and causes diastolic Ca^2+^ leakage from the sarcoplasmic reticulum (SR) (66, 67). MIF enhances both reverse-mode and forward-mode Na^+^/Ca^2+^ exchanger (NCX) activity, exacerbating intracellular Ca^2+^ fluctuations (58, 66). CaMKII-dependent phosphorylation of phospholamban (PLB) relieves PLB-mediated inhibition of SR Ca^2+^-ATPase (SERCA), thereby enhancing SR Ca^2+^ reuptake, increasing SR Ca^2+^ load, and augmenting Ca^2+^ transient amplitude (67). These opposing effects—diastolic leak combined with enhanced systolic reuptake—create a state of increased Ca^2+^ cycling flux, which predisposes to afterdepolarizations and maintains AF (66, 67). Concurrently, MIF activates the CXCR7-PI3K-AKT-eNOS signaling axis, promoting nitric oxide production and further disturbing Ca^2+^ homeostasis; CXCR7 alone appears sufficient to mediate this effect, as it forms a major receptor complex with CD74 in cardiomyocytes (67).

##### 3.3.1.2 Gap junction remodeling and connexin 43 (Cx43) downregulation

Gap junctions, primarily composed of connexin 43 (Cx43) in the atria, mediate electrical coupling between cardiomyocytes. Reduced Cx43 expression and its lateralized redistribution decrease conduction velocity and increase directionality, providing a substrate for multiple re-entrant waves (68). In left atrial tissue from AF patients, elevated MIF levels correlate with Cx43 downregulation and disrupted distribution (69). Mechanistically, recombinant MIF downregulates Cx43 in HL-1 cells via the ERK1/2 pathway (69). In a mouse myocardial infarction model, MIF knockout not only reduces inflammation but also prevents abnormal Cx43 redistribution (70); whether this direct effect occurs in atria without confounding inflammation remains to be determined. Furthermore, Cx43 knockdown itself suppresses calcium channel expression (Cav1.2, Cav3.1) and current densities (I_Ca,L, I_Ca,T), creating a positive feedback loop that exacerbates both electrical and structural remodeling (71).

#### 3.3.2 Atrial structural remodeling

The central feature of atrial structural remodeling is interstitial fibrosis, driven by an imbalance between excessive extracellular matrix (ECM) deposition and its degradation. This leads to atrial enlargement, reduced compliance, and disrupted electrical conduction homogeneity. MIF promotes structural remodeling through two interrelated mechanisms: activation of cardiac fibroblasts and dysregulation of ECM metabolism.

##### 3.3.2.1 Activation and proliferation of cardiac fibroblasts

Atrial fibroblasts are the primary effector cells in structural remodeling. Under normal conditions, they remain quiescent and produce only small amounts of collagen and ECM proteins. Under persistent stress, they transform into myofibroblasts and synthesize large quantities of ECM (72, 73). MIF acts as a pro-fibrotic factor: MIF overexpression increases atrial fibrosis in mice (66), whereas MIF knockout reduces fibrosis (74). A key marker of fibroblast activation is upregulation of α-SMA expression. MIF drives the transition of quiescent fibroblasts to an activated phenotype primarily through Src kinase signaling (74, 75). MIF also promotes cardiac fibroblast proliferation via the Src kinase pathway (75).

Intriguingly, while MIF promotes myofibroblast activation, its interaction with the soluble CD74 ectodomain (sCD74) can conversely trigger RIP1/RIP3-dependent necroptosis in these cells, providing a potential feedback mechanism for terminating the fibrotic response (76).

##### 3.3.2.2 ECM homeostasis imbalance and the fibrogenic network

Beyond fibroblast activation, MIF regulates the balance between ECM synthesis and degradation. In atrial tissue from AF patients and animal models, elevated MIF expression is associated with upregulation of type I collagen (Col-1), type III collagen (Col-3), matrix metalloproteinases (MMP-2, MMP-9), and TGF-β (74, 75). *In vitro* experiments confirm that recombinant MIF (rMIF) induces these fibrosis-related proteins in cardiac fibroblasts (74, 75). Although MMP-2 and MMP-9 mediate ECM degradation, their proteolytic activity reshapes the ECM microenvironment during early fibrosis, providing a structural foundation for cell migration and new matrix deposition, thereby driving fibrosis progression (77, 78).

MIF activates the TGF-β/Smad signaling axis by promoting Src-kinase-mediated phosphorylation of Smad2/3 (74). TGF-β then drives expression of pro-fibrotic genes, including connective tissue growth factor, collagens, and MMPs, via both Smad and non-Smad pathways (79). Furthermore, a positive feedback loop may exist between MIF and TGF-β. In atrial fibroblasts, MIF upregulates TGF-β expression (74, 75). In joint capsule fibroblasts, MIF induces TGF-β synthesis and release, and TGF-β in turn upregulates MIF expression (80). Whether this positive feedback loop operates in atrial fibroblasts has not yet been directly validated experimentally. Thus, MIF links Src-dependent fibroblast activation with TGF-β/Smad-mediated ECM dysregulation to drive atrial structural remodeling.

### 3.4 MIF and thrombogenesis in AF

Thromboembolism, particularly ischemic stroke, is a leading cause of death and disability in AF. Direct evidence linking MIF to AF-related thrombus formation is limited, but several studies in other disease settings suggest that MIF may promote a prothrombotic state by impairing endothelial function and activating the coagulation system. For example, in patients with spinal cord injury, each 1 ng/mL increase in plasma MIF is associated with an 11% higher risk of deep vein thrombosis (a venous thromboembolic event) (81); however, the relevance of this finding to AF-related arterial thromboembolism remains unclear, given the distinct pathophysiologies of venous and arterial thrombosis.

#### 3.4.1 Endothelial dysfunction

Vascular endothelial dysfunction—characterized by a shift from an anticoagulant, anti-inflammatory phenotype to a procoagulant, pro-inflammatory state—is an initiating step in AF-associated thrombus formation. MIF is a key driver of endothelial injury. In patients with end-stage renal disease, plasma MIF levels negatively correlate with endothelium-dependent vasodilation and positively correlate with arterial stiffness (82). Mechanistically, MIF induces the expression of intercellular adhesion molecule-1 (ICAM-1) and vascular cell adhesion molecule-1 (VCAM-1) in monocytes via the Src, PI3K, and NF-κB pathways, promoting monocyte adhesion to the endothelium and triggering endothelial inflammation and dysfunction (83). Consistently, MIF deficiency in endothelial cells reduces TNF-α-induced expression of multiple adhesion molecules and weakens leukocyte rolling and adhesion. Conversely, exogenous MIF cooperates with TNF-α to induce endothelial P-selectin expression (84).

#### 3.4.2 Enhanced procoagulant state

In addition to promoting endothelial dysfunction, MIF may directly enhance the procoagulant state. In patients with membranous nephropathy (a condition associated with nephrotic syndrome and hypercoagulability), plasma MIF levels positively correlate with platelet count and negatively correlate with prothrombin time, suggesting a hypercoagulable state (85); however, the mechanisms of hypercoagulability in nephrotic syndrome differ from those in AF, and direct evidence in AF patients is lacking. Platelets store and release MIF upon activation; secreted MIF then recruits inflammatory cells, delays thrombus retraction, and affects thrombus stability (86, 87). Furthermore, CD44—a co-receptor for MIF—is also involved in platelet function. Under high arterial shear stress, CD44-deficient mice exhibit enhanced platelet activation and thrombus formation (88), uggesting that CD44 signaling may inhibit excessive platelet activation. Since MIF signals through the CD74/CD44 complex, this raises the possibility that MIF might exert an anti-thrombotic effect via CD44 in some contexts. Thus, the net pro-thrombotic versus anti-thrombotic effect of MIF-CD44 signaling in AF-associated thrombus formation remains unclear and warrants further investigation in AF-specific models.

### 3.5 Potential association between MIF and the autonomic nervous system

Autonomic dysfunction, particularly sympathetic overactivation and vagal withdrawal, contributes to AF initiation and maintenance. Direct evidence linking MIF to autonomic remodeling in AF is currently lacking. However, indirect observations from non-AF models provide some clues.

In a myocardial infarction model, MIF knockout reduced susceptibility to ventricular arrhythmias without affecting sympathetic nerve regeneration or norepinephrine levels, suggesting that MIF’s pro-arrhythmic effect in that setting is primarily mediated by inflammation rather than direct neural modulation (70). Whether similar mechanisms operate in the atria of AF patients remains unknown. Central nervous system studies have shown that MIF can antagonize angiotensin II-induced sympathoexcitation in the paraventricular nucleus (PVN) via its intrinsic TPOR activity (89) and improve baroreflex function in the nucleus tractus solitarius (NTS) (90, 91). These findings come from hypertensive models, not AF, and their relevance to atrial autonomic regulation is uncertain.

Peripherally, MIF-driven macrophage polarization (e.g., via the HIF-1α/MIF/CD74 axis) exacerbates atrial remodeling (10, 70). Although this effect is attributed to inflammation rather than direct neural remodeling, inflammatory cytokines can influence autonomic nerve endings and ganglionic plexi, providing an indirect pathway by which MIF might modulate autonomic tone in AF. However, this hypothesis remains speculative.

Overall, current evidence does not support a direct role of MIF in autonomic nervous system regulation in AF. Nevertheless, the possibility that MIF indirectly influences autonomic function through inflammation-mediated pathways cannot be excluded and warrants future investigation in AF-specific experimental models.

## 4 Clinical evidence for MIF in AF

Circulating and tissue levels of MIF are closely associated with the onset, progression, and prognosis of AF. This association positions MIF not only as a key pathological mediator but also as a potential clinical biomarker. This section systematically reviews the clinical evidence regarding MIF in the diagnosis, classification, risk stratification, and prognosis of AF (summarized in **Table 1**). However, it should be noted that most clinical studies are observational, and the specific causal relationship between MIF and these diseases has not yet been established.

**Table 1:** Clinical studies of MIF in atrial fibrillation.

First author (Year)	Sample size	AF type	Main findings
Wan C (2018) (7)	186 AF patients, 103 healthy controls	Paroxysmal, persistent, permanent	Serum MIF significantly higher in AF patients than in healthy controls. Logistic regression showed MIF associated with AF. Permanent AF had highest MIF, followed by persistent, then paroxysmal. MIF positively correlated with left atrial diameter.
Bo Y K (2018) (8)	134 AF patients (29 paroxysmal, 51 persistent, 54 permanent), 54 healthy controls	Paroxysmal, persistent, permanent	Left atrial diameter, serum MIF, PICP, and PIIINP were significantly higher in AF patients than in controls (P < 0.05). MIF, PICP, and PIIINP levels increased progressively across AF subtypes (paroxysmal < persistent < permanent, P < 0.05). Left atrial diameter positively correlated with MIF (r=0.705, P < 0.01).
Keefe JA (2025) (9)	11 POAF patients, 6 sinus rhythm patients	Postoperative AF	Pericardial fluid MIF levels were significantly higher in poAF patients compared to SR patients 24–36 hours after cardiac surgery (1.7-fold, P = 0.038). Pericardial fluid from POAF patients induced STAT3 phosphorylation and IL-1β expression, blocked by MIF inhibitor.
Stoppe C (2013) (60)	46 patients undergoing elective coronary artery bypass grafting (25 on-pump, 21 off-pump)	Postoperative AF	MIF increased postoperatively only in conventional coronary artery bypass grafting patients (with I/R) (P = 0.024). Perioperative MIF release was associated with enhanced antioxidant capacity and significantly reduced postoperative incidence of AF (P = 0.018) and acute kidney injury (P = 0.048).
Pei X Y (2011) (92)	50 non-valvular AF patients, 50 healthy controls	Non-valvular AF	Serum MIF levels were significantly higher in AF patients (33.18 ± 6.07 ng/mL) than in controls (15.79 ± 5.16 ng/mL) (P<0.01). MIF levels positively correlated with left atrial diameter.
Ma X M (2024) (93)	175 chronic heart failure patients with AF, 100 chronic heart failure patients without AF	Chronic heart failure with AF	Serum MIF levels were higher in chronic heart failure patients with AF than in those without AF (P < 0.05). Among AF patients, the poor prognosis group (26.29% incidence at 6 months) had higher MIF levels (P < 0.05). Multivariate analysis showed elevated MIF as an independent risk factor for poor prognosis (all-cause death, heart failure rehospitalization, or stroke). ROC analysis showed AUC for MIF alone was 0.777 (95% CI 0.708–0.837, cutoff 252.47 pg/mL); combined with irisin and TMAO, AUC increased to 0.919 (95% CI 0.869–0.955).
Zhang C (2010) (94)	24 AF patients with rheumatic heart disease undergoing mitral valve replacement, 16 sinus rhythm controls	Rheumatic heart disease with AF	Plasma MIF levels were significantly higher in AF group than in SR group (P < 0.01). Myocardial MIF protein and mRNA expression were also higher in AF group. MIF expression positively correlated with AF duration.
Guo Q (2022) (95)	83 AF patients with ischemic stroke (45 paroxysmal, 38 persistent), 60 ischemic stroke controls, 50 healthy controls	Ischemic stroke with AF	Serum MIF levels were significantly higher in the AF-stroke group than in stroke controls and healthy controls (P < 0.01). Persistent AF patients had higher MIF than paroxysmal AF patients (P < 0.01). The poor prognosis group (mRS>3) had higher MIF levels (P < 0.01). MIF positively correlated with AF-ischemic stroke (r=0.224, P < 0.05). MIF was identified as an independent risk factor for AF with ischemic stroke by multivariate logistic regression (P < 0.05).
Leng F M (2023) (97)	280 hypertensive patients with AF (171 with left atrial low-voltage area<10%, 109 with left atrial low-voltage area≥10%)	Hypertension with AF	Serum MIF levels were significantly higher in the fibrosis group (left atrial low-voltage area≥10%) than in controls (P < 0.001). MIF positively correlated with atrial fibrosis markers: PINP (r=0.513), PICP (r=0.663), PIIINP (r=0.591), PCIII (r=0.643), and LN (r=0.585) (all P<0.05). Multivariate logistic regression identified MIF as an independent risk factor for left atrial fibrosis (P < 0.05).
Stoppe C (2012) (98)	52 patients undergoing elective cardiac surgery with cardiopulmonary bypass	Postoperative AF	Postoperative MIF levels were inversely correlated with organ dysfunction (SAPS II and SOFA scores) and positively correlated with cardiac power index. Compared to healthy volunteers, patients had elevated MIF levels before surgery (64 ± 50 vs. 13 ± 17 ng/mL, P < 0.05), with peak values at ICU admission.
Stoppe C (2015) (99)	100 patients undergoing elective cardiac surgery (coronary artery bypass grafting or valve replacement)	Postoperative AF	Intraoperative MIF levels were independently associated with reduced POAF risk (OR 0.99, 95% CI 0.98-1.00, P = 0.007). Circulating sCD74/MIF complexes detected in 50% of patients enhanced MIF antioxidant activity. High-expression MIF genotype was associated with reduced organ dysfunction incidence (3 vs. 25, P = 0.042).
Szabo T M (2025) (103)	70 HFrEF/HFmrEF patients (45.7% with AF/AFL)	HFrEF/HFmrEF patients (45.7% with AF/AFL)	MIF correlated negatively with LVEF (r=−0.33, P = 0.005) and TAPSE (r=−0.37, P = 0.001), and positively with LVGLS (r=0.41, P = 0.0004). High MIF (>520 pg/mL) was associated with higher prevalence of ischemic cardiomyopathy (57.1% vs. 28.6%, P = 0.029) and reduced 6-minute walk distance (324.8 vs. 404.0 m, P = 0.010). On multivariate analysis, MIF >520 pg/mL independently predicted low TAPSE (OR = 17.79, 95%CI 1.66–540.2, P = 0.041); the multivariate model achieved an AUC of 0.955 (95% CI 0.911–1.000).
Luedike P (2018) (104)	249 consecutive HF patients (both preserved and reduced EF)	Heart failure (not AF-specific)	MIF detectable in all, no difference between HF subtypes. MIF correlated with WBC (r=0.18, P = 0.005) and CRP (r=0.20, P = 0.003). MIF associated with PASP (r=0.23, P < 0.001); log-PASP independently associated with MIF on multivariable analysis (P = 0.02). At 180-day follow-up, higher MIF predicted all-cause mortality (HR = 1.01 per ng/mL, 95%CI 1.004–1.02, P = 0.005).
Omurzakova U (2025) (105)	120 patients with T2DM and/or HF (49 T2DM, 43 HF, 28 both)	T2DM and/or HF (not AF-specific)	12 weeks of empagliflozin treatment altered circulating levels of 12 proteins, including MIF. Proteomic analysis identified MIF as one of the differentially expressed proteins functionally linked to T2DM and/or HF pathophysiology, suggesting MIF may serve as a potential pharmacodynamic marker of SGLT2 inhibitor therapy.
Zhong Y F (2022) (106)	184 non-valvular AF patients (63 with ischemic stroke, 121 without stroke)	Non-valvular AF with ischemic stroke	Serum MIF levels were significantly higher in the stroke group than in the non-valvular AF group (P < 0.01). MIF positively correlated with CHA2DS2-VASc score (r=0.186, P = 0.012). Multivariate logistic regression identified MIF as an independent risk factor for non-valvular AF with ischemic stroke (P < 0.05).
Zhang L (2026) (107)	486 acute stroke patients (474 ischemic, 12 ICH; median follow-up >5 years)	Stroke (mixed etiologies, AF as major cause)	Higher acute-phase plasma MIF independently predicted recurrent stroke or TIA (adjusted HR per SD increment=1.56, 95%CI 1.18-2.06). Associations followed a dose-response pattern across MIF quartiles. Adding MIF to conventional risk factors + CRP improved 5-year recurrence prediction (ΔC-index 0.030-0.050).
Zhao Q (2019) (110)	498 STEMI patients post-PCI, 40 stable angina, 137 healthy controls	STEMI (not AF-specific)	Admission MIF 3- to 7-fold higher in STEMI vs controls (122 ± 61 vs 39 ± 19 vs 17 ± 8 ng/mL, P < 0.001). Higher admission MIF (≥127.8 ng/mL) independently predicted in-hospital mortality (OR = 9.1, 95%CI 1.7-47.2) and 3.2-year MACCE (HR = 2.8, 95%CI 1.5-5.6). AUC for in-hospital mortality 0.820.
Vyshnevska I (2022) (111)	134 STEMI patients undergoing primary PCI	STEMI (not AF-specific)	Pre-PCI MIF >3934 pg/mL independently predicted 1-year composite endpoint (all-cause death, non-fatal MI/stroke, unstable angina hospitalization, HF decompensation, urgent revascularization) with AUC = 0.7, sensitivity 54%, specificity 82% (P = 0.008). MIF correlated with WBC (r=0.33, P = 0.0001) and CRP (r=0.19, P = 0.032). Higher pre- and post-PCI MIF levels associated with adverse outcomes (OR = 1.0 each, both P < 0.05). MIF >3493 pg/mL predicted lower long-term survival (log-rank P = 0.00025).

### 4.1 MIF levels reflect AF burden and atrial remodeling

Case-control studies consistently show that circulating MIF levels in patients with AF are significantly higher than in sinus rhythm controls (7, 92). MIF levels exhibit a graded association with the clinical classification and disease burden of AF. Multiple studies have demonstrated that serum MIF concentrations increase progressively across AF types: paroxysmal < persistent < permanent (7, 8). This relationship has been validated across different patient populations. Among patients with chronic heart failure, those with AF have higher serum MIF levels than those without (93). In rheumatic heart disease patients undergoing valve replacement, plasma MIF levels are elevated in the AF group and positively correlate with AF duration (94). Similarly, in AF patients with ischemic stroke, MIF levels are markedly higher in persistent AF than in paroxysmal AF (95). Bioinformatics analyses further identify MIF as a potential key biomarker for AF (96). Together, these findings indicate that circulating MIF levels closely reflect both the clinical subtype and the chronicity of AF, serving as an objective biological indicator of overall disease burden and duration.

MIF levels are also closely associated with imaging and serological markers of atrial structural remodeling. At the imaging level, serum MIF positively correlates with left atrial diameter. A study of 134 AF patients reported a correlation coefficient of 0.705 between MIF and left atrial diameter; both parameters increased in parallel as AF progressed from paroxysmal to persistent to permanent (8). In hypertensive patients with AF, those with a left atrial low-voltage area ≥10% (indicating myocardial fibrosis) had significantly higher serum MIF levels than those with a low-voltage area <10% (97). At the serological level, MIF levels are positively associated with various markers of collagen synthesis and degradation, including the N-terminal propeptide of type I procollagen (PINP), the C-terminal propeptide of type I procollagen (PICP), the N-terminal propeptide of type III procollagen (PIIINP), type III procollagen (PCIII), and laminin (LN) (8, 97). Multivariate logistic regression analysis further confirmed that MIF is independently associated with left atrial low-voltage areas, a surrogate for atrial fibrosis (97). These findings link MIF to the pathological process of atrial structural remodeling and suggest that MIF may serve as a circulating biomarker for assessing the degree of atrial fibrosis.

### 4.2 The dual role of MIF in POAF

POAF is a major complication associated with prolonged hospitalization and adverse short- and long-term outcomes. MIF has gained considerable attention for its role in perioperative myocardial ischemia-reperfusion injury, but existing studies have yielded seemingly contradictory conclusions: some report cardioprotective effects (60, 98, 99), whereas others demonstrate a pro-arrhythmic action (9).

#### 4.2.1 Protective role

The protective role of MIF in POAF is observed in the early phase (minutes to hours after reperfusion), involves cardiomyocyte-derived MIF, and is reflected in peripheral blood samples. Oxidative stress is a major driver of POAF. Stoppe et al. found that in patients undergoing coronary artery bypass grafting with cardiopulmonary bypass, perioperative MIF levels were significantly elevated and positively correlated with endogenous superoxide dismutase activity and thioredoxin levels. Notably, patients with high MIF levels had a much lower incidence of POAF than those with low levels (8% versus 38%, *P* = 0.014) (60). Subsequent studies confirmed that peak MIF levels during the intraoperative reperfusion phase were independently and negatively associated with POAF risk (OR = 0.99, *P* = 0.007) and were associated with freedom from POAF (99).

Mechanistically, under I/R conditions, Cys81 of MIF undergoes S-nitrosylation, leading to an approximately twofold increase in its TPOR activity, which directly scavenges ROS (46). Additionally, MIF promotes glucose uptake in ischemic cardiomyocytes via the CD74/AMPK pathway, thereby reducing calcium overload and electrophysiological abnormalities (100). Overexpression of MIF reduces myocardial cell apoptosis and enhances autophagy by activating the ERK1/2 pathway, thereby mitigating I/R injury (101). Stoppe et al. also found that approximately half of the patients harbored a circulating complex of MIF and soluble CD74 (sCD74); this complex further enhanced MIF’s redox enzyme activity and attenuated H_2_O_2_-induced cardiomyocyte apoptosis *in vitro* (99). The critical role of MIF in endogenous cardioprotection is further underscored by the finding that genetic deletion of MIF completely abolishes the infarct-sparing effect of ischemic preconditioning (102).

#### 4.2.2 Pathogenic role

In contrast, the pathogenic role of MIF in POAF is observed in the subacute phase (24–72 hours postoperatively), involves myofibroblast-derived MIF, and is best reflected in pericardial fluid. Keefe et al. recently reported an apparently contrasting finding using single-cell RNA sequencing and a specific MIF inhibitor (9). They found that MIF in the atria of POAF mice primarily originates from *Acta2^+^* myofibroblasts rather than from cardiomyocytes. Treatment with the MIF inhibitor 4-IPP decreased the incidence of POAF to approximately one-fifth and shortened its duration to approximately one-ninth, while also reducing atrial macrophage infiltration. In pericardial fluid from cardiac surgery patients, MIF levels in those with POAF were 1.7-fold higher than in those who remained in sinus rhythm (24–36 hours postoperatively). Furthermore, pericardial fluid from POAF patients induced STAT3 phosphorylation and IL-1β expression in human THP-1 monocytes, an effect that could be blocked by MIF inhibitors (9).

#### 4.2.3 Reconciling the dual roles of MIF in POAF

The apparent contradiction between the protective and pathogenic roles of MIF in POAF may be attributed to differences in temporal dynamics, cellular origin, and sample source. First, immediately following I/R injury (minutes to hours after reperfusion), cardiomyocytes release pre-stored MIF, which directly protects the myocardium through its S-nitrosylation-enhanced antioxidant activity, thereby reducing oxidative stress-induced arrhythmias. During the postoperative subacute phase (24–72 hours), fibroblasts are activated by inflammatory signals and upregulate MIF expression, which amplifies local inflammation by recruiting macrophages and creates a pro-arrhythmic substrate. Second, protective MIF primarily originates from cardiomyocytes, with rapid, transcription-independent release; in contrast, deleterious MIF mainly stems from activated myofibroblasts, requires transcriptional upregulation, and peaks later. Single-cell data from Keefe et al. clearly demonstrate that MIF is highly expressed in Acta2+ fibroblasts (9), whereas MIF derived from cardiomyocytes has already declined at this stage. Furthermore, serum MIF reflects a systemic response and may include compensatory protective signals from other organs; in contrast, pericardial fluid directly reflects the local cardiac microenvironment and better represents the true MIF levels within the atrium. Thus, sample type is another key factor explaining the divergent findings.

### 4.3 Predictive value for long-term clinical outcomes

Heart failure, stroke, myocardial infarction, and death are the major adverse outcomes that define the long-term burden of AF. Emerging evidence indicates that circulating MIF levels hold independent predictive value for these events in patients with AF, supporting its candidacy as a prognostic biomarker. However, most available data come from observational studies with heterogeneous cutoff values, and dedicated evidence in pure AF cohorts remains limited.

#### 4.3.1 MIF and heart failure

A 6-month follow-up study of patients with chronic heart failure and AF demonstrated that those experiencing the composite endpoint of all-cause death, heart failure rehospitalization, or stroke (incidence 26.29%) had significantly higher baseline serum MIF levels. After multivariable adjustment, MIF remained an independent risk factor. The area under the curve (AUC) for MIF alone was 0.777; when combined with irisin and trimethylamine N-oxide, the AUC increased to 0.919 (93).

In heart failure with reduced or mildly reduced ejection fraction (HFrEF/HFmrEF), MIF levels correlate inversely with left ventricular ejection fraction (r = −0.33, *P* = 0.005) and tricuspid annular plane systolic excursion (TAPSE; r = −0.37, *P* = 0.001), and positively with left ventricular global longitudinal strain (r = 0.41, *P* = 0.0004). High MIF levels (>520 pg/mL) are associated with a higher prevalence of ischemic cardiomyopathy (57.1% versus 28.6%, *P* = 0.029) and reduced 6-minute walk distance (324.8 m versus 404.0 m, *P* = 0.010), indicating that elevated MIF reflects worse functional capacity (103). In a general heart failure cohort of 249 patients, MIF correlated with leukocyte count (r = 0.18, *P* = 0.005), C-reactive protein (r = 0.20, *P* = 0.003), and pulmonary artery systolic pressure (r = 0.23, *P* < 0.001), and was associated with all-cause death at 180 days (hazard ratio = 1.01, 95% CI: 1.004–1.02, *P* = 0.005) (104).

Beyond prognosis, MIF may also serve as a pharmacodynamic marker. In a prospective study of 120 patients with type 2 diabetes and/or heart failure, 12 weeks of empagliflozin treatment altered circulating levels of 12 proteins, including MIF, suggesting that MIF may participate in the cardioprotective mechanisms of SGLT2 inhibitors (105). However, this finding is preliminary and requires validation in dedicated AF cohorts.

#### 4.3.2 MIF and stroke

Ischemic stroke is the most devastating complication of AF. MIF levels are associated not only with stroke occurrence but also with post-stroke functional recovery and long-term recurrence risk. In patients with AF and ischemic stroke, multiple studies consistently demonstrate that MIF is closely associated with stroke occurrence and poor prognosis (95, 106). Serum MIF levels are significantly higher in AF patients with ischemic stroke than in those without a history of stroke. MIF levels also positively correlate with the CHA_2_DS_2_-VASc score, a stroke risk assessment tool. Regarding prognosis, patients with a poor outcome (defined as a modified Rankin Scale [mRS] score >3 at admission) have significantly higher serum MIF levels than those with a good outcome.

Beyond short-term outcomes, a prospective cohort study of 486 acute stroke patients (median follow-up >5 years) found that higher plasma MIF levels were independently associated with recurrent stroke or transient ischemic attack (adjusted hazard ratio = 1.56 per standard deviation increment, 95% CI: 1.18–2.06) and improved risk stratification for 5-year recurrence beyond conventional risk factors (ΔC-index 0.030–0.050) (107). Although this cohort included stroke patients of mixed etiologies, AF is a leading cause of recurrent stroke, making these findings relevant to AF-related cerebrovascular prognosis. In addition, MIF inhibitors may have potential in reducing post-stroke brain injury, as suggested by studies in cerebral ischemia models (108, 109); however, this remains speculative.

#### 4.3.3 MIF and myocardial infarction

Myocardial infarction (MI) is both a frequent comorbidity in AF patients and a major contributor to adverse cardiovascular outcomes. The prognostic value of MIF in acute MI, particularly ST-segment elevation myocardial infarction (STEMI), has been substantiated in independent cohorts.

In a large cohort of 498 STEMI patients treated with primary percutaneous coronary intervention (PCI), admission MIF levels were 3- to 7-fold higher than in stable angina or control groups. A higher admission MIF level (≥127.8 ng/mL) was independently associated with in-hospital mortality (OR = 9.1, 95% CI: 1.7–47.2) and long-term major adverse cardio- and/or cerebrovascular events (MACCE) over 3.2 years of follow-up (hazard ratio = 2.8, 95% CI: 1.5–5.6), with an AUC of 0.820 for in-hospital mortality. Notably, MIF provided predictive value independent of high-sensitivity troponin T, NT-proBNP, and the Global Registry of Acute Coronary Events (GRACE) score (110). Another study of 134 STEMI patients reported that a pre-PCI MIF level >3934 pg/mL was independently associated with a composite endpoint (all-cause death, non-fatal MI, non-fatal stroke, unstable angina hospitalization, heart failure decompensation, and urgent revascularization) at 1-year follow-up (AUC = 0.7, 95% CI 0.578–0.753, sensitivity 54%, specificity 82%). Kaplan-Meier analysis showed that patients with MIF >3493 pg/mL had significantly lower long-term survival (log-rank *P* = 0.00025). Both pre- and post-PCI MIF levels were associated with adverse outcomes (OR = 1.0, 95% CI 1.0001–1.0008, *P* = 0.013; and OR = 1.0, 95% CI 1.0001–1.0009, *P* = 0.019, respectively) (111).

Although these investigations enrolled STEMI patients rather than AF-specific cohorts, their relevance to AF-related prognosis is supported by epidemiological data showing that AF complicates 11–15% of acute MI cases and independently increases 1-year case fatality (adjusted OR = 1.47, 95% CI 1.07–2.01) (112). Therefore, MIF’s robust prognostic value in STEMI likely extends to AF patients who develop acute coronary events, although dedicated studies in this specific population are warranted.

#### 4.3.4 Summary and limitations

Collectively, circulating MIF levels are associated with heart failure, stroke, and myocardial infarction—the three major adverse outcomes of AF. In some studies, MIF improves risk prediction beyond traditional clinical factors and may emerge as a response marker for therapies such as SGLT2 inhibitors. However, it should be emphasized that all clinical associations reported in Section 4 are derived from observational studies; causality has not been established. Evidence in pure AF cohorts is sparse for outcomes such as myocardial infarction, with most data originating from broader cardiovascular populations. Moreover, several methodological issues need to be addressed for clinical translation. First, serum and plasma MIF levels may exhibit systematic differences, and the lack of uniform sample types across studies affects the comparability of results. Second, the currently used assay protocols or reagents lack standardization, leading to discrepancies in absolute quantitative results between laboratories. Finally, the MIF cutoff values adopted in existing studies are highly heterogeneous, significant range differences, with no unified consensus. Future efforts should focus on standardizing detection protocols and validating uniform clinical decision thresholds in large-scale, multicenter prospective cohorts.

## 5 MIF-targeted therapies

MIF possesses multiple pharmacologically targetable domains, including the CALC redox center, the Pro1 tautomerase active site, and the Cys81 redox switch that controls the transition to oxMIF. Based on these structural features, current MIF-targeted strategies fall into three categories: (1) small-molecule inhibitors targeting the tautomerase active site, including the reversible competitive inhibitor ISO-1 and the irreversible covalent inhibitor 4-IPP; (2) receptor-level blockers that disrupt MIF-CD74/CD44 or chemokine receptor interactions; and (3) isoform-selective antibodies targeting oxMIF. Other MIF tautomerase inhibitors (e.g., TE-11, Z-312) (113, 114), a MIF-CD74 protein interaction blocker (MIF098) (115, 116), a PROTAC degrader (MD13) (117), and a clinical-stage MIF inhibitor (IPG1094, ClinicalTrials.gov identifiers NCT06212076, NCT05112159) have been reported in non-AF contexts, but none have been tested in AF models (summarized in **Table 2**). Although some of these agents have demonstrated therapeutic potential in inflammatory diseases and cancer, their application in AF remains in early exploratory stages. This section reviews MIF-targeted strategies and their progress in AF research.

**Table 2:** Summary of MIF-targeting agents.

Type	Name	Binding site	Mechanism	Advantages	Disadvantages
MIF inhibitor	ISO-1	MIF tautomerase active site (reversible binding)	Reversible inhibitor: competitively binds to the catalytic active site, inhibits enzyme activity and downstream signaling.	Well-established as a research tool, commonly used in proof-of-concept experiments.	Relatively low inhibitory activity (IC50 ~7 µM), may require higher doses for *in vivo* applications (118).
MIF inhibitor	4-IPP	MIF tautomerase active site (covalent modification)	Suicide substrate/irreversible inhibitor: irreversibly covalently binds to the critical proline (N-terminal Proline) at the catalytic active site, permanently inactivating MIF protein.	5–10 times more potent than ISO-1; irreversible mechanism provides longer duration of action.	As an irreversible inhibitor, higher risk of off-target effects, which may introduce uncertainty (123).
MIF inhibitor	TE-11	MIF tautomerase active site	Reversible inhibitor: inhibits MIF tautomerase activity (IC50 = 5.63 µM), reduces leukocyte migration, prevents M1 macrophage polarization.	Reduces mucosal damage and inflammation score in colitis model; classical mechanism.	Moderate inhibitory activity; only validated in inflammatory bowel disease, completely unexplored in cardiovascular disease (113).
MIF inhibitor	Z-312	MIF tautomerase active site (covalent/slow-tight binding)	High-activity tautomerase inhibitor (IC50 = 0.55 µM), inhibits microglial inflammatory cytokine production, blocks NF-κB and MAPK pathways.	High inhibitory activity (0.55 µM), superior to ISO-1; clear neuroprotective effects (Parkinson’s disease model).	Only validated in neuroinflammation/neurodegenerative diseases; completely unexplored in cardiovascular disease; exact binding site awaits co-crystal validation (114).
MIF inhibitor	p425	Allosteric site between MIF trimers (interface binding)	Allosteric inhibitor: binds to the unique interface formed by two MIF trimers, covering the tautomerase and allosteric pockets, exerting allosteric effects.	Unique mechanism of action; superior potency in inhibiting MIF-CD74 binding (IC50 = 0.40 µM) compared to ISO-1.	High molecular weight (904.9 Da), poor drug-like properties, not suitable for direct drug development (144).
MIF inhibitor	IPG1094	MIF tautomerase active site (highly selective)	First MIF small-molecule inhibitor to enter clinical trials; inhibits myeloid-derived suppressor cell formation, restores CD8^+^ T cell-mediated anti-tumor immunity.	Earliest clinical-stage agent (Phase I completed, Phase II ongoing); good safety profile (no >Grade 2 adverse events in Phase I); oral administration, high selectivity.	Entirely focused on oncology (lung cancer brain metastases); cardiovascular field unexplored; no studies on atrial fibrillation-related electrical remodeling or fibrosis.
MIF inhibitor	Ibudilast (MN-166)	MIF allosteric site (trimer interface); PDE4, PDE3A/B, PDE10, PDE11 catalytic sites	Multi−target drug: MIF allosteric inhibitor; non−selective PDE inhibitor (primarily inhibits PDE4 with ~30−fold weaker inhibition of PDE3A/B). Approved in Japan for bronchial asthma and cerebrovascular diseases.	Clinically mature (approved in Japan), oral administration, known safety profile; unique allosteric inhibition mechanism.	MIF inhibition is not specific (also inhibits PDE); no targeted validation in cardiovascular disease (cerebrovascular use exists, but not atrial fibrillation); weak PDE3 inhibition carries a theoretical risk of positive inotropy and pro−arrhythmic effects, although no clear cardiovascular adverse events have been observed in clinical studies (145).
MIF inhibitor	MIF098	MIF protein surface (binding site unknown)	MIF/CD74 interaction inhibitor: directly binds to MIF protein, alters its conformation or steric hindrance, blocks its binding to CD74 receptor, thereby inhibiting downstream signaling.	Relatively high selectivity, mainly blocks the pathological MIF-CD74 signaling axis, preserves other physiological functions.	Exact binding site unclear, making drug design and optimization difficult; relatively high dose in animal studies (115, 116).
Receptor antagonist	Anti-CD74 antibody	CD74 receptor	Blocks MIF-CD74 binding.	Most direct validation in atrial fibrillation; clear mechanism.	Biologic agent, high cost, requires injection (66).
Receptor antagonist	Anti-CD44 antibody	CD44 receptor	Blocks MIF signaling complex.	Anti-fibrotic and anti-oxidative.	Target not sufficiently specific; association with MIF in atrial fibrillation needs further clarification (125, 126).
Receptor antagonist	SB225002	CXCR2	Competitive antagonist of CXCR2.	Can reverse established atrial fibrillation; high selectivity.	Not MIF-specific; may affect other ligands (127, 128).
Receptor antagonist	AMD3100	CXCR4	Competitive antagonist of CXCR4.	Clinically approved, high translational potential.	Not MIF-specific; broad physiological functions (129).
OxMIF antibody	Imalumab (BAX69)	oxMIF	First-generation humanized monoclonal antibody, neutralizes oxMIF activity.	Completed Phase I/II clinical trials, validated target safety and preliminary anti-tumor efficacy.	Short half-life in humans; prone to aggregation; poor pharmacokinetic properties; limits use in chronic diseases (135).
OxMIF antibody	ON203	oxMIF	Second-generation engineered monoclonal antibody, neutralizes oxMIF; enhances antibody-dependent cell-mediated cytotoxicity via Fc region mutations.	Longer half-life, lower aggregation tendency; significantly better anti-tumor efficacy than Imalumab; lower off-target risk; modulates tumor microenvironment.	Still in preclinical stage, lacks human clinical trial data (136).
OxMIF antibody	ON104	oxMIF	Next-generation monoclonal antibody, specifically neutralizes oxMIF without triggering immune effector functions (no antibody-dependent cell-mediated cytotoxicity/complement-dependent cytotoxicity ).	Precise neutralization, high safety; effective in chronic inflammation models (glomerulonephritis, rheumatoid arthritis, colitis); suitable for long-term administration.	Preclinical stage; human efficacy and safety need validation (137, 138).
OxMIF inhibitor	PAV-174	oxMIF	Small molecule compound, inhibits oxMIF activity; also inhibits HSV-1 replication and tau protein phosphorylation/aggregation.	Dual effects: antiviral and anti-tau pathology; provides new mechanism for Alzheimer’s disease.	Preclinical stage; in vivo safety, dosage, and efficacy unknown (139).
PROTAC	MD13	MIF protein (cereblon E3 ligase)	PROTAC degrader: degrades MIF via ubiquitin-proteasome pathway (DC50 ≈ 100 nM, Dmax = 92%), eliminates all functions of MIF.	Extremely high degradation efficiency, almost completely clears MIF; novel mechanism of action (first MIF-targeting PROTAC).	Only validated in oncology (lung cancer); completely unexplored in cardiovascular disease; large molecular weight, pharmacokinetic challenges, unknown off-target degradation risk (117).

### 5.1 MIF small-molecule inhibitors

#### 5.1.1 ISO-1

ISO-1 is a reversible small-molecule inhibitor of MIF tautomerase activity (118). In cardiovascular research, ISO-1 has shown context-dependent effects. It attenuates high-glucose- or MIF-induced cardiomyocyte apoptosis *in vitro* (119, 120) and improves cardiac function and survival in septic shock rats (121). However, in a mouse model of metabolic syndrome, ISO-1 unexpectedly worsened cardiac dysfunction by blocking compensatory mitochondrial biogenesis (122). These discordant findings underscore the complex, context-dependent biology of MIF and the potential risk of non-selective inhibition. To date, ISO-1 has not been tested in any AF model, and its poor oral bioavailability limits chronic application. Given the absence of AF-specific data and the potential for adverse effects observed in other disease contexts, ISO-1 is not a viable candidate for AF therapy. Nevertheless, the lessons learned from its contradictory effects highlight the need for more selective MIF-targeting strategies.

#### 5.1.2 4-IPP

4-IPP is an irreversible MIF inhibitor that acts as a suicide substrate, covalently modifies the N-terminal proline residue, with an inhibitory potency approximately 5- to 10-fold higher than that of ISO-1 (123). In a mouse model of POAF, 4-IPP (50 mg/kg/d) significantly reduced AF incidence and duration; this effect was attributed to decreased atrial macrophage infiltration and reduced pro-inflammatory cytokine expression (9). Thus, 4-IPP demonstrates clear antiarrhythmic efficacy in the POAF setting.

As an irreversible inhibitor targeting Pro1, 4-IPP blocks MIF’s tautomerase activity and its pro-inflammatory functions. However, the potential impact of 4-IPP on MIF’s TPOR activity—which is mediated by the CALC motif (124) and contributes to cardioprotection via Cys81 S-nitrosation (46)—remains unknown and requires direct experimental validation. Thus, as a pan-MIF inhibitor, 4-IPP may simultaneously block both the protective (antioxidant) and pathogenic (pro-inflammatory) functions of MIF, raising concerns about unintended consequences. Therefore, non-selective MIF inhibition with 4-IPP may entail a trade-off between benefit and risk.

To translate 4-IPP into AF therapy, future studies should define a therapeutic time window (e.g., delayed administration until 12–24 h post-surgery to preserve early cardioprotective MIF signaling) or explore whether co-administration with antioxidants (e.g., N-acetylcysteine) could mitigate the potential oxidative risk resulting from inhibition of MIF’s antioxidant activity without compromising anti-inflammatory efficacy. Addressing these issues will be essential for the rational translation of 4-IPP into AF clinical practice.

### 5.2 Receptor-targeting strategies

#### 5.2.1 Targeting the MIF receptor complex: anti-CD74 and anti-CD44 antibodies

MIF signaling requires the formation of a receptor complex involving CD74 and CD44. Neutralizing anti-CD74 antibodies attenuate MIF-induced calcium dysregulation, electrophysiological abnormalities, atrial fibrosis, and premature atrial beats in AF models (66). Similarly, anti-CD44 blocking antibodies reduce AF inducibility in TGF-β transgenic mice, an effect associated with decreased STAT3 activation and collagen expression (125). In CD44-knockout mice, atrial tachypacing-induced oxidative stress, CaMKII/RyR2 abnormalities, and AF susceptibility are significantly reduced (126). These findings suggest that disrupting the MIF-CD74-CD44 signaling axis at the receptor level may represent a promising anti-AF strategy. Targeting the MIF receptor complex exhibits clear antiarrhythmic and anti-remodeling effects in preclinical models.

However, the safety of targeting CD74 or CD44 requires careful evaluation, given their critical roles in immune function (CD74 participates in major histocompatibility complex class II antigen presentation and B-cell maturation; CD44 mediates cell adhesion and migration). Systemic blockade of these receptors may lead to unintended immunosuppression, underscoring the need for atrial-specific or locally delivered therapeutic approaches. Thus, the major challenge for clinical translation of this strategy is to achieve local atrial efficacy while avoiding systemic immunosuppression.

#### 5.2.2 Chemokine receptor antagonists

Since MIF exerts some of its effects through chemokine receptors (e.g., CXCR2 and CXCR4), targeting these downstream receptors can indirectly block the pathological actions of MIF. In angiotensin II-infused mice, the CXCR2 inhibitor SB225002, along with CXCR2 knockout or bone marrow-specific CXCR2 deletion, significantly reduced AF inducibility, atrial fibrosis, macrophage infiltration, and superoxide production (127). The consistency of findings across pharmacological and genetic interventions strengthens the conclusion that CXCR2 is a key mediator of MIF-induced atrial remodeling. These data identify CXCR2 as a critical downstream mediator of MIF-driven atrial remodeling.

In spontaneously hypertensive rats, SB225002 not only prevented but also reversed pre-existing AF development, accompanied by reduced blood pressure, atrial remodeling, inflammation, oxidative stress, and conduction abnormalities. These effects were associated with suppression of multiple signaling pathways, including TGF-β/Smad2/3, NF-κB, NOX1/2, and restoration of ion channel (Kir2.1, Kv1.5) and gap junction (Cx43) expression (128). Additionally, the CXCR4 antagonist AMD3100 has been tested in a CaCl_2_-acetylcholine-induced AF mouse model. AMD3100 reduced AF incidence and duration by blocking the CXCL12/CXCR4 signaling axis, decreasing atrial inflammation and fibrosis. Mechanistically, AMD3100 reduced the recruitment of CD3+ T lymphocytes and F4/80+ macrophages, and suppressed overactivation of the ERK1/2 and AKT/mTOR pathways (129). Hence, indirect targeting of chemokine receptors may offer a more refined intervention, but its impact on MIF-mediated protective signaling requires validation.

Although SB225002 and AMD3100 do not directly target MIF, they represent indirect strategies to block downstream MIF-CXCR2/CXCR4 signaling. Whether these approaches offer advantages over direct MIF inhibition—for example, by preserving MIF’s antioxidant functions—remains to be determined and warrants further investigation. However, studies in myocardial I/R models caution that CXCR2 also mediates potentially protective MIF signaling in cardiomyocytes, while driving detrimental inflammation via infiltrating leukocytes (130). Such compartmentalized actions imply that systemic CXCR2 antagonism could inadvertently ablate endogenous cardioprotection. Future AF therapies may therefore require cell-selective targeting or time-restricted regimens to mitigate this risk. In summary, successful application of chemokine receptor antagonists in AF will require a careful balance between anti-inflammatory efficacy and preservation of cardioprotection.

### 5.3 Antibodies targeting OxMIF: a hypothesis-generating perspective

Under oxidative stress, MIF undergoes a conformational change to form a distinct isoform, termed oxMIF. MIF exists in two redox-dependent conformational isoforms, of which oxMIF is selectively expressed on the plasma membrane and immune cell surfaces of patients with inflammatory diseases but is barely detectable in healthy individuals, defining it as a disease-associated conformational isoform (131). Structural biology studies have further confirmed that under oxidative conditions, MIF adopts a modified and more dynamic structure, suggesting that this redox-dependent conformational switch may serve as an important regulator of MIF function (132). Mechanistically, under inflammatory conditions, myeloperoxidase released from neutrophils produces hypochlorous acid, which efficiently oxidizes MIF to oxMIF. This myeloperoxidase-driven oxidation induces conformational changes that promote the binding of oxMIF to its receptor CD74—a capability that native MIF lacks (133). Therefore, targeting oxMIF offers higher disease selectivity and a wider therapeutic window compared with non-selective MIF inhibition (134), which is particularly attractive for chronic inflammatory conditions such as AF. Thus, oxMIF represents an ideal target that combines disease specificity with a favorable therapeutic window.

Several anti-oxMIF antibodies have been developed. Imalumab (BAX69), a first-generation humanized anti-oxMIF monoclonal antibody, has entered Phase I clinical trials for cancer (ClinicalTrials.gov identifier: NCT01765790) but exhibits a short half-life, aggregation tendency, and unfavorable pharmacokinetics (135). To overcome these limitations, ON203, a second-generation antibody, has shown improved anti-tumor efficacy with lower off-target risk (136). For chronic inflammatory diseases, ON104 was engineered to specifically neutralize oxMIF without triggering immune cell effector functions and has demonstrated efficacy in preclinical models of glomerulonephritis and rheumatoid arthritis (137, 138). More recently, PAV-174, a novel small molecule targeting oxMIF, has been reported to inhibit oxMIF-induced tau phosphorylation and aggregation in Alzheimer’s disease models, further illustrating the versatility of oxMIF-targeted strategies (139). Although these agents have shown promise in oncology and inflammatory diseases, none have been evaluated in AF.

Given that AF is driven by inflammation and oxidative stress, oxMIF represents a promising but entirely unexplored therapeutic target in this context. To date, however, no study has examined oxMIF expression in atrial tissue or plasma from AF patients, and none of the oxMIF-targeting antibodies have been tested in AF models. Future studies should first validate oxMIF expression in atrial tissue and plasma of AF patients, particularly in subsets with high inflammatory burden (e.g., POAF or obesity-related AF). If confirmed, oxMIF-targeting antibodies or small molecules could provide a disease-selective strategy to avoid the dual (protective and pathogenic) effects associated with non-selective MIF inhibition. Subsequent preclinical studies in AF animal models will be essential to establish proof-of-concept for this approach. In conclusion, oxMIF-targeted strategies hold promises for precision intervention, but their value in AF urgently awaits experimental validation.

## 6 Limitations and future perspectives

This review has systematically integrated current evidence on MIF in AF. MIF promotes AF through multiple mechanisms: activation of pro-inflammatory NF-κB signaling and M1 macrophage polarization (Section 3.1); contribution to oxidative stress alongside antioxidant protection via its TPOR activity (Section 3.2); disruption of ion channels, calcium homeostasis, and gap junctions leading to electrical remodeling (Section 3.3.1); and activation of fibroblasts and the TGF-β/Smad axis driving structural remodeling (Section 3.3.2). Clinically, circulating MIF levels correlate with AF burden, atrial fibrosis, and long-term outcomes including heart failure, stroke, and myocardial infarction (Section 4). Therapeutically, several agents—including the irreversible inhibitor 4-IPP, receptor-level blockers (anti-CD74/CD44 antibodies, CXCR2/CXCR4 antagonists), and other compounds (ISO-1, TE-11, Z-312, MIF098, MD13, IPG1094, anti-oxMIF antibodies)—have shown efficacy or potential in various models, although only 4-IPP and the receptor blockers have been tested in AF models (Section 5).

### 6.1 Limitations

Despite these advances, several key challenges and unresolved issues remain. First, opposing effects of MIF in the heart. Acute, cardiomyocyte-derived MIF protects against oxidative stress via its TPOR activity (mediated by the CALC motif and enhanced by Cys81 S-nitrosylation), whereas persistent or fibroblast-derived MIF promotes inflammation, fibrosis, and AF. The molecular switch that determines this functional divergence remains unknown. Potential modulators include the local redox environment, the nature of the upstream stimulus (hypoxia versus mechanical stretch), and the cell type (cardiomyocyte, fibroblast, or macrophage). Identifying this switch is critical for developing selective therapies.

Second, lack of causal evidence. The vast majority of clinical studies are cross-sectional or case-control in design. They can only demonstrate associations between circulating MIF levels and AF parameters, not causality. It remains unclear whether elevated MIF drives AF pathogenesis or merely reflects the inflammatory response secondary to AF. Residual confounding (e.g., baseline systemic inflammation, renal function, temporal changes in atrial size) may bias estimates of “independent” associations. Furthermore, there are no prospective interventional studies evaluating whether MIF-guided risk stratification or MIF-targeted therapy improves AF outcomes.

Third, non-selectivity of current inhibitors. Although 4-IPP, anti-CD74/CD44 antibodies, and CXCR2/CXCR4 antagonists have shown efficacy in AF models, all are non-selective to varying degrees. 4-IPP blocks the tautomerase active site (Pro1) but its impact on TPOR activity remains unknown. Receptor-level blockers may interfere with physiological functions of CD74 (major histocompatibility complex class II antigen presentation) and CD44 (cell adhesion). Highly selective strategies, such as anti-oxMIF antibodies, have not yet been tested in any AF model. Moreover, the optimal therapeutic time window (e.g., delayed administration to preserve early cardioprotection) has not been defined.

Fourth, AF heterogeneity. AF is a heterogeneous syndrome; the contribution of MIF likely differs among valvular, non-valvular, lone, and POAF, yet no studies have stratified by AF subtype.

### 6.2 Future perspectives

Despite the progress summarized in this review, several critical questions remain to be addressed before MIF-targeted strategies can be translated to the bedside. From a mechanistic standpoint, it will be essential to dissect how the local microenvironment—particularly the redox state and prevailing inflammatory tone—dictates whether MIF exerts protective or pathogenic actions in the atrium. The putative positive feedback loop between MIF and TGF-β, which has been functionally confirmed in joint capsule fibroblasts, awaits direct validation in atrial fibroblasts. In parallel, the application of single-cell and spatial transcriptomics to human atrial tissue across different AF subtypes holds considerable promise for resolving cell-type-specific MIF expression patterns and their relationship to disease severity. On the causal inference front, the observational nature of current clinical evidence necessitates approaches that can mitigate confounding and reverse causation. Mendelian randomization offers one such approach, leveraging genetic variants as instrumental variables to infer causality. Although a large-scale Mendelian randomization study using cis-pQTLs found no causal relationship between genetically predicted total MIF levels and AF (140), existing Mendelian randomization results are inconclusive and may be confounded by the fact that circulating total MIF, as measured in genome-wide association study, does not capture the functionally relevant fraction—oxMIF. Given that MIF oxidation is driven by the local redox environment, which itself is shaped by disease activity, a positive feedback loop exists that conventional Mendelian randomization cannot easily resolve. Future MR studies should aim to instrument oxMIF specifically, potentially by identifying cis-pQTLs for oxMIF using recently developed oxMIF-specific antibody-based proteomic platforms in large cohorts. In parallel, two-step Mendelian randomization could be employed to test whether genetically determined oxidative stress markers (e.g., urate, glutathione peroxidase) modify the effect of MIF on AF risk, consistent with a redox-dependent conversion model. In addition, despite the well-documented associations of MIF promoter polymorphisms (rs755622 and rs5844572) with other cardiovascular diseases (Section 2.1), their role in AF remains completely unexplored. Future studies should therefore investigate whether these functional variants are associated with AF susceptibility, disease progression, or response to therapy. Prospective cohorts stratified by MIF genotype would further define the incremental prognostic value of MIF beyond established risk indices like CHA_2_DS_2_-VASc.

The refinement of therapeutic strategies will require careful consideration of both target selectivity and timing. Given the dual roles of MIF in the heart, targeting the disease-associated conformational isoform oxMIF represents a particularly attractive avenue, as it could selectively neutralize pathogenic MIF in inflamed atrial tissue while preserving the antioxidant and cardioprotective functions of the native protein. Validation of oxMIF expression in atrial samples and plasma from AF patients—especially those with inflammation-driven subtypes such as postoperative, obesity-related, or obstructive sleep apnea-associated AF—is a prerequisite for testing oxMIF-directed antibodies (e.g., ON104) in preclinical AF models. In the specific context of POAF, a time-window-based intervention may prove critical. The available evidence suggests that MIF inhibition should be avoided during the first 6–12 hours following I/R to safeguard endogenous cardioprotection, whereas delayed inhibition initiated during the subacute phase (24–72 hours post-surgery) could suppress the pro-arrhythmic inflammatory cascade driven by fibroblast-derived MIF. This concept requires rigorous validation in models directly comparing early versus delayed administration of inhibitors such as 4-IPP.

Finally, the inherent heterogeneity of AF demands that future preclinical and clinical investigations move beyond a one-size-fits-all approach and instead stratify patients by AF subtype. MIF-directed therapies are most likely to yield benefit in conditions where inflammation is a dominant driver, and may have limited efficacy in purely structural forms of the arrhythmia such as long-standing valvular AF. While drug repurposing efforts with agents like ibudilast or IPG1094 offer a potentially accelerated clinical path, their evaluation in AF-specific models remains a prerequisite. Looking beyond the pathways detailed in this review, emerging evidence suggests that MIF also regulates fundamental cellular processes including autophagy induction (141), metabolic reprogramming of immune cells (142), and stem cell niche maintenance (143). Whether these broader biological functions of MIF actively contribute to the development or perpetuation of the atrial substrate represents an intriguing frontier for future exploration. Addressing these interconnected priorities will be essential to fully harness the therapeutic potential of targeting MIF in atrial fibrillation.

## References

[1] Cheng S He J Han Y Han S Li P Liao H . Global burden of atrial fibrillation/atrial flutter and its attributable risk factors from 1990 to 2021. Europace. (2024) 26:euae195. doi: 10.1093/europace/euae195 38984719 PMC11287210

[2] Lloyd-Jones DM Wang TJ Leip EP Larson MG Levy D Vasan RS . Lifetime risk for development of atrial fibrillation: the Framingham Heart Study. Circulation. (2004) 110:1042–6. doi: 10.1161/01.Cir.0000140263.20897.42 15313941

[3] Karatela MF Calkins H . The global impact of atrial fibrillation. Arrhythm Electrophysiol Rev. (2025) 14:e28. doi: 10.15420/aer.2025.33 41346409 PMC12673497

[4] Ren H Lai H Chen Z . Inflammatory and fibrotic signaling pathways mediated by cardiac macrophages in atrial fibrillation. Front Cardiovasc Med. (2025) 12:1692638. doi: 10.3389/fcvm.2025.1692638 41561130 PMC12812952

[5] Zhou X Dudley SCJ . Evidence for inflammation as a driver of atrial fibrillation. Front Cardiovasc Med. (2020) 7:62. doi: 10.3389/fcvm.2020.00062 32411723 PMC7201086

[6] Aliyarbayova A Sultanova T Yaqubova S Najafova T Sadiqova G Salimova A . Macrophage migration inhibitory factor: its multifaceted role in inflammation and immune regulation across organ systems. Cell Physiol Biochem. (2025) 59:569–88. doi: 10.33594/000000809 40916855

[7] Wan C Li Z . Serum macrophage migration inhibitory factor is correlated with atrial fibrillation. J Clin Lab Anal. (2018) 32:e22225. doi: 10.1002/jcla.22225 28407372 PMC6817180

[8] Bo YK . Correlation between macrophage migration inhibitors and atrial remodeling in atrial fibrillation. Urumqi: Xinjiang Medical University (2020). doi: 10.27433/d.cnki.gxyku.2020.000731

[9] Keefe JA Navarro-Garcia JA Zhao S Chelu MG Wehrens XH . Atrial fibroblast-derived macrophage migration inhibitory factor promotes atrial macrophage accumulation in postoperative atrial fibrillation. JCI Insight. (2025) 10:e190756. doi: 10.1172/jci.insight.190756 40811033 PMC12487863

[10] He H Zhou Z Zhang L Lu Z Li B Li X . HIF1α/MIF/CD74 signaling mediated OSA-induced atrial fibrillation by promoting M1 macrophages polarization. Int Immunopharmacol. (2025) 149:114248. doi: 10.1016/j.intimp.2025.114248 39929098

[11] Bloom BR Bennett B . Mechanism of a reaction *in vitro* associated with delayed-type hypersensitivity. Science. (1966) 153:80–2. doi: 10.1126/science.153.3731.80 5938421

[12] David JR . Delayed hypersensitivity *in vitro*: its mediation by cell-free substances formed by lymphoid cell-antigen interaction. Proc Natl Acad Sci USA. (1966) 56:72–7. doi: 10.1073/pnas.56.1.72 5229858 PMC285677

[13] Calandra T Bernhagen J Mitchell RA Bucala R . The macrophage is an important and previously unrecognized source of macrophage migration inhibitory factor. J Exp Med. (1994) 179:1895–902. doi: 10.1084/jem.179.6.1895 8195715 PMC2191507

[14] Calandra T Bernhagen J Metz CN Spiegel LA Bacher M Donnelly T . MIF as a glucocorticoid-induced modulator of cytokine production. Nature. (1995) 377:68–71. doi: 10.1038/377068a0 7659164

[15] Bernhagen J Calandra T Mitchell RA Martin SB Tracey KJ Voelter W . MIF is a pituitary-derived cytokine that potentiates lethal endotoxaemia. Nature. (1993) 365:756–9. doi: 10.1038/365756a0 8413654

[16] Burger-Kentischer A Göbel H Kleemann R Zernecke A Bucala R Leng L . Reduction of the aortic inflammatory response in spontaneous atherosclerosis by blockade of macrophage migration inhibitory factor (MIF). Atherosclerosis. (2006) 184:28–38. doi: 10.1016/j.atherosclerosis.2005.03.028 15921687

[17] Schmeisser A Marquetant R Illmer T Graffy C Garlichs CD Böckler D . The expression of macrophage migration inhibitory factor 1alpha (MIF 1alpha) in human atherosclerotic plaques is induced by different proatherogenic stimuli and associated with plaque instability. Atherosclerosis. (2005) 178:83–94. doi: 10.1016/j.atherosclerosis.2004.08.038 15585204

[18] Calandra T Roger T . Macrophage migration inhibitory factor: a regulator of innate immunity. Nat Rev Immunol. (2003) 3:791–800. doi: 10.1038/nri1200 14502271 PMC7097468

[19] Budarf M McDonald T Sellinger B Kozak C Graham C Wistow G . Localization of the human gene for macrophage migration inhibitory factor (MIF) to chromosome 22q11.2. Genomics. (1997) 39:235–6. doi: 10.1006/geno.1996.4505 9027512

[20] Paralkar V Wistow G . Cloning the human gene for macrophage migration inhibitory factor (MIF). Genomics. (1994) 19:48–51. doi: 10.1006/geno.1994.1011 8188240

[21] Sun HW Bernhagen J Bucala R Lolis E . Crystal structure at 2.6-A resolution of human macrophage migration inhibitory factor. Proc Natl Acad Sci USA. (1996) 93:5191–6. doi: 10.1073/pnas.93.11.5191 8643551 PMC39220

[22] Li YY Wang H Zhang YY . Macrophage migration inhibitory factor gene rs755622 G/C polymorphism and coronary artery disease: a meta-analysis of 8,488 participants. Front Cardiovasc Med. (2022) 9:959028. doi: 10.3389/fcvm.2022.959028 36186991 PMC9515403

[23] Du GL Luo JY Wang D Li YH Fang BB Li XM . MIF gene rs755622 polymorphism positively associated with acute coronary syndrome in Chinese Han population: case-control study. Sci Rep. (2020) 10:140. doi: 10.1038/s41598-019-56949-z 31924846 PMC6954175

[24] El-Mahdy RI Saleem TH Essam OM Algowhary M . Functional variants in the promoter region of macrophage migration inhibitory factor rs755622 gene (MIF G173C) among patients with heart failure: association with echocardiographic indices and disease severity. Heart Lung. (2021) 50:92–100. doi: 10.1016/j.hrtlng.2020.07.015 32800392

[25] Lan MY Chang YY Chen WH Tseng YL Lin HS Lai SL . Association between MIF gene polymorphisms and carotid artery atherosclerosis. Biochem Biophys Res Commun. (2013) 435:319–22. doi: 10.1016/j.bbrc.2013.02.129 23537651

[26] Valdés-Alvarado E Muñoz-Valle JF Valle Y Sandoval-Pinto E García-González IJ Valdez-Haro A . Association between the -794 (CATT)5-8 MIF gene polymorphism and susceptibility to acute coronary syndrome in a western Mexican population. J Immunol Res. (2014) 2014:704854. doi: 10.1155/2014/704854 25105152 PMC4106097

[27] Leng L Metz CN Fang Y Xu J Donnelly S Baugh J . MIF signal transduction initiated by binding to CD74. J Exp Med. (2003) 197:1467–76. doi: 10.1084/jem.20030286 12782713 PMC2193907

[28] Shi X Leng L Wang T Wang W Du X Li J . CD44 is the signaling component of the macrophage migration inhibitory factor-CD74 receptor complex. Immunity. (2006) 25:595–606. doi: 10.1016/j.immuni.2006.08.020 17045821 PMC3707630

[29] Potru PS Spittau B . CD74: a prospective marker for reactive microglia? Neural Regener Res. (2023) 18:2673–4. doi: 10.4103/1673-5374.371350 37449617 PMC10358643

[30] Bernhagen J Krohn R Lue H Gregory JL Zernecke A Koenen RR . MIF is a noncognate ligand of CXC chemokine receptors in inflammatory and atherogenic cell recruitment. Nat Med. (2007) 13:587–96. doi: 10.1038/nm1567 17435771

[31] Alampour-Rajabi S El Bounkari O Rot A Müller-Newen G Schober A Weber C . MIF interacts with CXCR7 to promote receptor internalization, ERK1/2 and ZAP-70 signaling, and lymphocyte chemotaxis. FASEB J. (2015) 29:4497–511. doi: 10.1096/fj.15-273904 26139098

[32] Jankauskas SS Wong DWL Bucala R Djudjaj S Boor P . Evolving complexity of MIF signaling. Cell Signal. (2019) 57:76–88. doi: 10.1016/j.cellsig.2019.01.006 30682543

[33] Subbannayya T Variar P Advani J Nair B Shankar S Gowda H . An integrated signal transduction network of macrophage migration inhibitory factor. J Cell Commun Signal. (2016) 10:165–70. doi: 10.1007/s12079-016-0326-x 27139435 PMC4882307

[34] Calandra T Bucala R . Macrophage migration inhibitory factor (MIF): a glucocorticoid counter-regulator within the immune system. Crit Rev Immunol. (1997) 17:77–88. doi: 10.1615/critrevimmunol.v17.i1.30 9034724

[35] Rassaf T Weber C Bernhagen J . Macrophage migration inhibitory factor in myocardial ischaemia/reperfusion injury. Cardiovasc Res. (2014) 102:321–8. doi: 10.1093/cvr/cvu071 24675723

[36] Zhao L Zhao BH Ruze A Li QL Deng AX Gao XM . Distinct roles of MIF in the pathogenesis of ischemic heart disease. Cytokine Growth Factor Rev. (2024) 80:121–37. doi: 10.1016/j.cytogfr.2024.10.005 39438226

[37] Pressley KR Naseem Y Nalawade S Forsthuber TG . The distinct functions of MIF in inflammatory cardiomyopathy. Front Immunol. (2025) 16:1544484. doi: 10.3389/fimmu.2025.1544484 40092999 PMC11906721

[38] Youness RA Elemam NM Abdelhamid AM Mohamed AH Elsherbiny LM Ramzy A . Macrophage migration inhibitory factor (MIF) and the tumor ecosystem: a tale of inflammation, immune escape, and tumor growth. Front Immunol. (2025) 16:1636839. doi: 10.3389/fimmu.2025.1636839 41159021 PMC12554771

[39] Morrison MC Kleemann R . Role of macrophage migration inhibitory factor in obesity, insulin resistance, type 2 diabetes, and associated hepatic co-morbidities: a comprehensive review of human and rodent studies. Front Immunol. (2015) 6:308. doi: 10.3389/fimmu.2015.00308 26124760 PMC4467247

[40] Vasella M Wolf S Francis EC Grieb G Pfister P Reid G . Involvement of the macrophage migration inhibitory factor (MIF) in lipedema. Metabolites. (2023) 13:1105. doi: 10.3390/metabo13101105 37887430 PMC10608777

[41] Matejuk A Benedek G Bucala R Matejuk S Offner H Vandenbark AA . MIF contribution to progressive brain diseases. J Neuroinflamm. (2024) 21:8. doi: 10.1186/s12974-023-02993-6 38178143 PMC10765708

[42] Breidung D Megas IF Freytag DL Bernhagen J Grieb G . The role of macrophage migration inhibitory factor (MIF) and D-dopachrome tautomerase (D-DT/MIF-2) in infections: a clinical perspective. Biomedicines. (2023) 12:2. doi: 10.3390/biomedicines12010002 38275363 PMC10813530

[43] Schindler L Dickerhof N Hampton MB Bernhagen J . Post-translational regulation of macrophage migration inhibitory factor: basis for functional fine-tuning. Redox Biol. (2018) 15:135–42. doi: 10.1016/j.redox.2017.11.028 29247897 PMC5975065

[44] Kleemann R Kapurniotu A Frank RW Gessner A Mischke R Flieger O . Disulfide analysis reveals a role for macrophage migration inhibitory factor (MIF) as thiol-protein oxidoreductase. J Mol Biol. (1998) 280:85–102. doi: 10.1006/jmbi.1998.1864 9653033

[45] Kleemann R Kapurniotu A Mischke R Held J Bernhagen J . Characterization of catalytic centre mutants of macrophage migration inhibitory factor (MIF) and comparison to Cys81Ser MIF. Eur J Biochem. (1999) 261:753–66. doi: 10.1046/j.1432-1327.1999.00327.x 10215893

[46] Luedike P Hendgen-Cotta UB Sobierajski J Totzeck M Reeh M Dewor M . Cardioprotection through S-nitros(yl)ation of macrophage migration inhibitory factor. Circulation. (2012) 125:1880–9. doi: 10.1161/circulationaha.111.069104 22415145

[47] SChinagl A Kerschbaumer RJ Sabarth N Douillard P Scholz P Voelkel D . Role of the cysteine 81 residue of macrophage migration inhibitory factor as a molecular redox switch. Biochemistry. (2018) 57:1523–32. doi: 10.1021/acs.biochem.7b01156 29412660

[48] Kimura H Sato Y Tajima Y Suzuki H Yukitake H Imaeda T . BTZO-1, a cardioprotective agent, reveals that macrophage migration inhibitory factor regulates ARE-mediated gene expression. Chem Biol. (2010) 17:1282–94. doi: 10.1016/j.chembiol.2010.10.011 21168764

[49] Yukitake H Takizawa M Kimura H . Macrophage migration inhibitory factor as an emerging drug target to regulate antioxidant response element system. Oxid Med Cell Longev. (2017) 2017:8584930. doi: 10.1155/2017/8584930 28191280 PMC5278225

[50] Mathew B Jacobson JR Siegler JH Moitra J Blasco M Xie L . Role of migratory inhibition factor in age-related susceptibility to radiation lung injury via NF-E2-related factor-2 and antioxidant regulation. Am J Respir Cell Mol Biol. (2013) 49:269–78. doi: 10.1165/rcmb.2012-0291OC 23526214 PMC3824032

[51] Aviles RJ Martin DO Apperson-Hansen C Houghtaling PL Rautaharju P Kronmal RA . Inflammation as a risk factor for atrial fibrillation. Circulation. (2003) 108:3006–10. doi: 10.1161/01.Cir.0000103131.70301.4f 14623805

[52] Leftheriotis DI Fountoulaki KT Flevari PG Parissis JT Panou FK Andreadou IT . The predictive value of inflammatory and oxidative markers following the successful cardioversion of persistent lone atrial fibrillation. Int J Cardiol. (2009) 135:361–9. doi: 10.1016/j.ijcard.2008.04.012 18640731

[53] Guo Y Lip GY Apostolakis S . Inflammation in atrial fibrillation. J Am Coll Cardiol. (2012) 60:2263–70. doi: 10.1016/j.jacc.2012.04.063 23194937

[54] Ihara K Sasano T . Role of inflammation in the pathogenesis of atrial fibrillation. Front Physiol. (2022) 13:862164. doi: 10.3389/fphys.2022.862164 35492601 PMC9047861

[55] Naveed H Ishaque A Nadeem A Hussain Rathore AW Akhtar S Haroon S . Pathogenesis of oxidative stress biomarkers in atrial fibrillation: a narrative review. J Innov Card Rhythm Manag. (2026) 17(1):6591–606. doi: 10.19102/icrm.2026.17012 41657713 PMC12880196

[56] Rao F Deng CY Zhang QH Xue YM Xiao DZ Kuang SJ . Involvement of Src tyrosine kinase and protein kinase C in the expression of macrophage migration inhibitory factor induced by H2O2 in HL-1 mouse cardiac muscle cells. Braz J Med Biol Res. (2013) 46:746–51. doi: 10.1590/1414-431x20132936 24036910 PMC3854426

[57] Takahashi M Nishihira J Shimpo M Mizue Y Ueno S Mano H . Macrophage migration inhibitory factor as a redox-sensitive cytokine in cardiac myocytes. Cardiovasc Res. (2001) 52:438–45. doi: 10.1016/s0008-6363(01)00408-4 11738060

[58] Chin CG Chen YC Lin YK Lu YY Cheng WL Chung CC . Effect of macrophage migration inhibitory factor on pulmonary vein arrhythmogenesis through late sodium current. Europace. (2023) 25:698–706. doi: 10.1093/europace/euac152 36056883 PMC10103572

[59] Chuang YC Su WH Lei HY Lin YS Liu HS Chang CP . Macrophage migration inhibitory factor induces autophagy via reactive oxygen species generation. PloS One. (2012) 7:e37613. doi: 10.1371/journal.pone.0037613 22629429 PMC3358253

[60] Stoppe C Werker T Rossaint R Dollo F Lue H Wonisch W . What is the significance of perioperative release of macrophage migration inhibitory factor in cardiac surgery? Antioxid Redox Signal. (2013) 19:231–9. doi: 10.1089/ars.2012.5015 23157710 PMC3691912

[61] Pohl J Hendgen-Cotta UB Rammos C Luedike P Mull E Stoppe C . Targeted intracellular accumulation of macrophage migration inhibitory factor in the reperfused heart mediates cardioprotection. Thromb Haemost. (2016) 115:200–12. doi: 10.1160/th15-05-0436 26310191

[62] Koga K Kenessey A Powell SR Sison CP Miller EJ Ojamaa K . Macrophage migration inhibitory factor provides cardioprotection during ischemia/reperfusion by reducing oxidative stress. Antioxid Redox Signal. (2011) 14:1191–202. doi: 10.1089/ars.2010.3163 20831446

[63] Koga K Kenessey A Ojamaa K . Macrophage migration inhibitory factor antagonizes pressure overload-induced cardiac hypertrophy. Am J Physiol Heart Circ Physiol. (2013) 304:H282–93. doi: 10.1152/ajpheart.00595.2012 23144312

[64] Rao F Deng CY Wu SL Xiao DZ Yu XY Kuang SJ . Involvement of Src in L-type Ca2+ channel depression induced by macrophage migration inhibitory factor in atrial myocytes. J Mol Cell Cardiol. (2009) 47:586–94. doi: 10.1016/j.yjmcc.2009.08.030 19744492

[65] Rao F Deng CY Wu SL Xiao DZ Huang W Deng H . Mechanism of macrophage migration inhibitory factor-induced decrease of T-type Ca(2+) channel current in atrium-derived cells. Exp Physiol. (2013) 98:172–82. doi: 10.1113/expphysiol.2012.066761 22848081

[66] Cheng WL Kao YH Chen YC Lin YK Chen SA Chen YJ . Macrophage migration inhibitory factor increases atrial arrhythmogenesis through CD74 signaling. Transl Res. (2020) 216:43–56. doi: 10.1016/j.trsl.2019.10.002 31669150

[67] Tang Z Liu F Nishi M Mackay F Harada M Takeshima H . Macrophage migration inhibitory factor induces phospholamban phosphorylation in cardiac muscle. Cell Calcium. (2025) 130:103051. doi: 10.1016/j.ceca.2025.103051 40680416

[68] Guo YH Yang YQ . Atrial fibrillation: focus on myocardial connexins and gap junctions. Biol (Basel). (2022) 11:489. doi: 10.3390/biology11040489 35453689 PMC9029470

[69] Li X Rao F Deng CY Wei W Liu FZ Yang H . Involvement of ERK1/2 in Cx43 depression induced by macrophage migration inhibitory factor in atrial myocytes. Clin Exp Pharmacol Physiol. (2017) 44:771–8. doi: 10.1111/1440-1681.12766 28429502

[70] Lyu J Huang J Wu J Yu T Wei X Lei Q . Lack of macrophage migration inhibitory factor reduces susceptibility to ventricular arrhythmias during the acute phase of myocardial infarction. J Inflammation Res. (2021) 14:1297–311. doi: 10.2147/jir.S304553 33854357 PMC8039209

[71] Peng DW Lai YY Luo XS Li X Deng CY Guo HM . Connexin 43 participates in atrial electrical remodelling through colocalization with calcium channels in atrial myocytes. Clin Exp Pharmacol Physiol. (2022) 49:25–34. doi: 10.1111/1440-1681.13580 34438468

[72] Pakshir P Noskovicova N Lodyga M Son DO Schuster R Goodwin A . The myofibroblast at a glance. J Cell Sci. (2020) 133:jcs227900. doi: 10.1242/jcs.227900 32651236

[73] Yue L Xie J Nattel S . Molecular determinants of cardiac fibroblast electrical function and therapeutic implications for atrial fibrillation. Cardiovasc Res. (2011) 89:744–53. doi: 10.1093/cvr/cvq329 20962103 PMC3039247

[74] Wang HL Li ZQ Liu F Fang BB Fan P . Macrophage migration inhibitory factor promotes the proliferation and activation of atrial fibroblasts through the Src kinase signaling pathway. Chin J Cell Biol. (2025) 47(10):2465–74. doi: 10.11844/cjcb.2025.10.0003

[75] Xue YM Deng CY Wei W Liu FZ Yang H Liu Y . Macrophage migration inhibitory factor promotes cardiac fibroblast proliferation through the Src kinase signaling pathway. Mol Med Rep. (2018) 17:3425–31. doi: 10.3892/mmr.2017.8261 29257298

[76] Soppert J Kraemer S Beckers C Averdunk L Möllmann J Denecke B . Soluble CD74 reroutes MIF/CXCR4/AKT-mediated survival of cardiac myofibroblasts to necroptosis. J Am Heart Assoc. (2018) 7:e009384. doi: 10.1161/jaha.118.009384 30371153 PMC6201423

[77] Kong YZ Yu X Tang JJ Ouyang X Huang XR Fingerle-Rowson G . Macrophage migration inhibitory factor induces MMP-9 expression: implications for destabilization of human atherosclerotic plaques. Atherosclerosis. (2005) 178:207–15. doi: 10.1016/j.atherosclerosis.2004.08.030 15585220

[78] Pakozdi A Amin MA Haas CS Martinez RJ Haines GK 3rd Santos LL . Macrophage migration inhibitory factor: a mediator of matrix metalloproteinase-2 production in rheumatoid arthritis. Arthritis Res Ther. (2006) 8:R132. doi: 10.1186/ar2021 16872482 PMC1779381

[79] Chen F Lyu L Xing C Chen Y Hu S Wang M . The pivotal role of TGF-β/Smad pathway in fibrosis pathogenesis and treatment. Front Oncol. (2025) 15:1649179. doi: 10.3389/fonc.2025.1649179 40969268 PMC12440922

[80] Zhang Y Liu Z Wang K Lu S Fan S Xu L . Macrophage migration inhibitory factor regulates joint capsule fibrosis by promoting TGF-β1 production in fibroblasts. Int J Biol Sci. (2021) 17:1837–50. doi: 10.7150/ijbs.57025 33994866 PMC8120472

[81] Wu DM Zheng ZH Wang S Wen X Han XR Wang YJ . Association between plasma macrophage migration inhibitor factor and deep vein thrombosis in patients with spinal cord injuries. Aging (Albany NY). (2019) 11:2447–56. doi: 10.18632/aging.101935 31036774 PMC6520010

[82] Rammos C Hendgen-Cotta UB Sobierajski J Adamczyk S Hetzel GR Kleophas W . Macrophage migration inhibitory factor is associated with vascular dysfunction in patients with end-stage renal disease. Int J Cardiol. (2013) 168:5249–56. doi: 10.1016/j.ijcard.2013.08.021 23978362

[83] Amin MA Haas CS Zhu K Mansfield PJ Kim MJ Lackowski NP . Migration inhibitory factor up-regulates vascular cell adhesion molecule-1 and intercellular adhesion molecule-1 via Src, PI3 kinase, and NFkappaB. Blood. (2006) 107:2252–61. doi: 10.1182/blood-2005-05-2011 16317091 PMC1472703

[84] Cheng Q McKeown SJ Santos L Santiago FS Khachigian LM Morand EF . Macrophage migration inhibitory factor increases leukocyte-endothelial interactions in human endothelial cells via promotion of expression of adhesion molecules. J Immunol. (2010) 185:1238–47. doi: 10.4049/jimmunol.0904104 20554956

[85] Ding N Li PL Wu KL Lv TG Yu WL Hao J . Macrophage migration inhibitory factor levels are associated with disease activity and possible complications in membranous nephropathy. Sci Rep. (2022) 12:18558. doi: 10.1038/s41598-022-23440-1 36329091 PMC9633699

[86] Strüßmann T Tillmann S Wirtz T Bucala R von Hundelshausen P Bernhagen J . Platelets are a previously unrecognised source of MIF. Thromb Haemost. (2013) 110:1004–13. doi: 10.1160/th13-01-0049 23846621

[87] Wirtz TH Tillmann S Strüßmann T Kraemer S Heemskerk JW Grottke O . Platelet-derived MIF: a novel platelet chemokine with distinct recruitment properties. Atherosclerosis. (2015) 239:1–10. doi: 10.1016/j.atherosclerosis.2014.12.039 25561410

[88] Liu G Liu G Alzoubi K Chatterjee M Walker B Münzer P . CD44 sensitivity of platelet activation, membrane scrambling and adhesion under high arterial shear rates. Thromb Haemost. (2016) 115:99–108. doi: 10.1160/th14-10-0847 26355696

[89] Colombari E Colombari DS Li H Shi P Dong Y Jiang N . Macrophage migration inhibitory factor in the paraventricular nucleus plays a major role in the sympathoexcitatory response to salt. Hypertension. (2010) 56:956–63. doi: 10.1161/hypertensionaha.110.155101 20937969 PMC3130992

[90] Freiria-Oliveira AH Blanch GT Li H Colombari E Colombari DS Sumners C . Macrophage migration inhibitory factor in the nucleus of solitary tract decreases blood pressure in SHRs. Cardiovasc Res. (2013) 97:153–60. doi: 10.1093/cvr/cvs297 22997157 PMC3584959

[91] Barbosa RM Speretta GF Dias DPM Ruchaya PJ Li H Menani JV . Increased expression of macrophage migration inhibitory factor in the nucleus of the solitary tract attenuates renovascular hypertension in rats. Am J Hypertens. (2017) 30:435–43. doi: 10.1093/ajh/hpx001 28158469 PMC5861587

[92] Pei XY Hu XS Kuang BJ Liu W Yan W Min M . The concentrations and significance of plasma human macrophage migration inhibitory factor in non-valvular atrial fibrillation patients. Guide China Med. (2011) 9:177–8. doi: 10.15912/j.cnki.gocm.2011.19.114

[93] Ma XM Li J Li WP Wang LZ Liu Y An JY . Study on the relationship between serum irisin, TMAO, MIF and the prognosis of patients with chronic heart failure and atrial fibrillation and its predictive value. Prog Mod BioMed. (2024) 24:2145–2149+2129. doi: 10.13241/j.cnki.pmb.2024.11.027

[94] Zhang C . The Roles of Interleukin (Il)-18 and Macrophage Migration Inhibitory Factor (Mif) in Atrial Fibrillation. Xi'an: Air Force Medical University (2010). doi: 10.7666/d.d219391

[95] Guo Q Zhang Y Li HJ Gao S . Correlation analysis of microRNA-221, macrophage migration inhibitory factor, thrombomodulin and atrial fibrillation complicated with ischemic stroke. Hainan Med J. (2022) 33:2321–5. doi: 10.3969/j.issn.1003-6350.2022.18.003

[96] Wu X Yan X Ma R Wu Q Pan X Wu Q . Ion channels and atrial fibrillation: mitophagy as a key mediator. Front Physiol. (2025) 16:1687578. doi: 10.3389/fphys.2025.1687578 41357167 PMC12675238

[97] Leng FM Li FY Lu C Xing X . Correlation between serum macrophage migration inhibitor levels and atrial fibrosis in patients with hypertension and atrial fibrillation. Chin J Hypertens. (2023) 31:668–72. doi: 10.16439/j.issn.1673-7245.2023.07.012

[98] Stoppe C Grieb G Rossaint R Simons D Coburn M Götzenich A . High postoperative blood levels of macrophage migration inhibitory factor are associated with less organ dysfunction in patients after cardiac surgery. Mol Med. (2012) 18:843–50. doi: 10.2119/molmed.2012.00071 22526918 PMC3409286

[99] Stoppe C Rex S Goetzenich A Kraemer S Emontzpohl C Soppert J . Interaction of MIF family proteins in myocardial ischemia/reperfusion damage and their influence on clinical outcome of cardiac surgery patients. Antioxid Redox Signal. (2015) 23:865–79. doi: 10.1089/ars.2014.6243 26234719 PMC4615780

[100] Miller EJ Li J Leng L McDonald C Atsumi T Bucala R . Macrophage migration inhibitory factor stimulates AMP-activated protein kinase in the ischaemic heart. Nature. (2008) 451:578–82. doi: 10.1038/nature06504 18235500

[101] Chen XC Gai MT He CH Zhao BH Liu F Ma X . Recombinant dsAAV9-mediated endogenous overexpression of macrophage migration inhibitory factor alleviates myocardial ischemia-reperfusion injury via activating AMPK and ERK1/2 signaling pathways. Cardiovasc Drugs Ther. (2025) 39:1243–57. doi: 10.1007/s10557-024-07662-1 39747743

[102] Ruze A Chen BD Liu F Chen XC Gai MT Li XM . Macrophage migration inhibitory factor plays an essential role in ischemic preconditioning-mediated cardioprotection. Clin Sci (Lond). (2019) 133:665–80. doi: 10.1042/cs20181013 30804219

[103] Szabo TM Vass M Germán-Salló M Frigy A Nagy EE . Elevated macrophage migration inhibitory factor 1 is associated with left and right ventricular systolic dysfunction in heart failure with reduced ejection fraction. Biomedicines. (2025) 13:1087. doi: 10.3390/biomedicines13051087 40426915 PMC12108681

[104] Luedike P Alatzides G Papathanasiou M Heisler M Pohl J Lehmann N . Circulating macrophage migration inhibitory factor (MIF) in patients with heart failure. Cytokine. (2018) 110:104–9. doi: 10.1016/j.cyto.2018.04.033 29723777

[105] Omurzakova U Breidert M Donner M Toktogulova N Moldobaeva M Quartier A . Proteomic signatures and blood adenosine triphosphate levels as markers of empagliflozin efficacy in type 2 diabetes mellitus and heart failure. Horm Metab Res. (2025) 57:653–61. doi: 10.1055/a-2716-7109 41248679

[106] Zhong YF Lei CY Li YM Yuan F Zhang XM . Relationship of miR-221, MIF and sTM with AF plus ischemic stroke. Chin J Geriatr Heart Brain Vessel Dis. (2022) 24:273–6. doi: 10.3969/j.issn.1009-0126.2022.03.013

[107] Zhang L Antabi MA Mattar J El Bounkari O Fang R Waegemann K . Circulating cytokine levels and 5-year vascular recurrence after stroke: a multicenter prospective cohort study. Eur Stroke J. (2026) 11:23969873251360145. doi: 10.1093/esj/23969873251360145 40790506 PMC12343552

[108] Ji W Ren Y Wei X Ding X Dong Y Yuan B . Ischemic stroke protected by ISO-1 inhibition of apoptosis via mitochondrial pathway. Sci Rep. (2023) 13:2788. doi: 10.1038/s41598-023-29907-z 36797398 PMC9935850

[109] Liu YC Tsai YH Tang SC Liou HC Kang KH Liou HH . Cytokine MIF enhances blood-brain barrier permeability: impact for therapy in ischemic stroke. Sci Rep. (2018) 8:743. doi: 10.1038/s41598-017-16927-9 29335619 PMC5768806

[110] Zhao Q Men L Li XM Liu F Shan CF Zhou XR . Circulating MIF levels predict clinical outcomes in patients with ST-elevation myocardial infarction after percutaneous coronary intervention. Can J Cardiol. (2019) 35:1366–76. doi: 10.1016/j.cjca.2019.04.028 31495686

[111] Vyshnevska I Storozhenko T Kopytsya M Bila N . Prediction of one-year adverse clinical outcomes by macrophage migration inhibitory factor in stemi patients. EUREKA: Health Sci. (2022), 19–29. doi: 10.21303/2504-5679.2022.002714

[112] Bengtson LG Chen LY Chamberlain AM Michos ED Whitsel EA Lutsey PL . Temporal trends in the occurrence and outcomes of atrial fibrillation in patients with acute myocardial infarction (from the Atherosclerosis Risk in Communities Surveillance Study). Am J Cardiol. (2014) 114:692–7. doi: 10.1016/j.amjcard.2014.05.059 25048343 PMC4609175

[113] Vámos E Vántus VB Deák P Kálmán N Sturm EM Nayak BB . MIF tautomerase inhibitor TE-11 prevents inflammatory macrophage activation and glycolytic reprogramming while reducing leukocyte migration and improving Crohn's disease-like colitis in male mice. Front Immunol. (2025) 16:1558079. doi: 10.3389/fimmu.2025.1558079 40330457 PMC12053165

[114] Zheng LT Chen J Zhang L Zhang Y Xu L Hou T . Inhibition of neuroinflammation by MIF inhibitor 3-({[4-(4-methoxyphenyl)-6-methyl-2-pyrimidinyl]thio}1methyl)benzoic acid (Z-312). Int Immunopharmacol. (2021) 98:107868. doi: 10.1016/j.intimp.2021.107868 34153665

[115] Huang H Chen D Pu J Yuan A Fu Q Li J . The small molecule macrophage migration inhibitory factor antagonist MIF098, inhibits pulmonary hypertension associated with murine SLE. Int Immunopharmacol. (2019) 76:105874. doi: 10.1016/j.intimp.2019.105874 31499270

[116] Shin MS Kang Y Wahl ER Park HJ Lazova R Leng L . Macrophage migration inhibitory factor regulates U1 small nuclear RNP immune complex-mediated activation of the NLRP3 inflammasome. Arthritis Rheumatol. (2019) 71:109–20. doi: 10.1002/art.40672 30009530 PMC6310104

[117] Xiao Z Song S Chen D van Merkerk R van der Wouden PE Cool RH . Proteolysis targeting chimera (PROTAC) for macrophage migration inhibitory factor (MIF) has anti-proliferative activity in lung cancer cells. Angew Chem Int Ed Engl. (2021) 60:17514–21. doi: 10.1002/anie.202101864 34018657 PMC8362126

[118] Lubetsky JB Dios A Han J Aljabari B Ruzsicska B Mitchell R . The tautomerase active site of macrophage migration inhibitory factor is a potential target for discovery of novel anti-inflammatory agents. J Biol Chem. (2002) 277:24976–82. doi: 10.1074/jbc.M203220200 11997397

[119] Dhanantwari P Nadaraj S Kenessey A Chowdhury D Al-Abed Y Miller EJ . Macrophage migration inhibitory factor induces cardiomyocyte apoptosis. Biochem Biophys Res Commun. (2008) 371:298–303. doi: 10.1016/j.bbrc.2008.04.070 18439909 PMC3104268

[120] Liang JL Xiao DZ Liu XY Lin QX Shan ZX Zhu JN . High glucose induces apoptosis in AC16 human cardiomyocytes via macrophage migration inhibitory factor and c-Jun N-terminal kinase. Clin Exp Pharmacol Physiol. (2010) 37:969–73. doi: 10.1111/j.1440-1681.2010.05420.x 20573157

[121] Wang FZ Jing L Chen J Huang YY . Role of macrophage migration inhibitory factor in septic shock-induced cardiovascular dysfunction: experiment with rats. Zhonghua Yi Xue Za Zhi. (2007) 87:768–73. doi: 10.3760/j:issn:0376-2491.2007.11.012 17565848

[122] Palud A Marciniak C Montaigne D Marechal X Ballot C Hassoun SM . Macrophage migration inhibitory factor inhibition is deleterious for high-fat diet-induced cardiac dysfunction. PloS One. (2013) 8:e58718. doi: 10.1371/journal.pone.0058718 23536817 PMC3594150

[123] Winner M Meier J Zierow S Rendon BE Crichlow GV Riggs R . A novel, macrophage migration inhibitory factor suicide substrate inhibits motility and growth of lung cancer cells. Cancer Res. (2008) 68:7253–7. doi: 10.1158/0008-5472.Can-07-6227 18794110 PMC2726006

[124] Thiele M Bernhagen J . Link between macrophage migration inhibitory factor and cellular redox regulation. Antioxid Redox Signal. (2005) 7:1234–48. doi: 10.1089/ars.2005.7.1234 16115028

[125] Chang SH Yeh YH Lee JL Hsu YJ Kuo CT Chen WJ . Transforming growth factor-β-mediated CD44/STAT3 signaling contributes to the development of atrial fibrosis and fibrillation. Basic Res Cardiol. (2017) 112:58. doi: 10.1007/s00395-017-0647-9 28871329

[126] Chen WJ Chang SH Chan YH Lee JL Lai YJ Chang GJ . Tachycardia-induced CD44/NOX4 signaling is involved in the development of atrial remodeling. J Mol Cell Cardiol. (2019) 135:67–78. doi: 10.1016/j.yjmcc.2019.08.006 31419440

[127] Zhang YL Cao HJ Han X Teng F Chen C Yang J . Chemokine receptor CXCR-2 initiates atrial fibrillation by triggering monocyte mobilization in mice. Hypertension. (2020) 76:381–92. doi: 10.1161/hypertensionaha.120.14698 32639881

[128] Zhang YL Teng F Han X Li PB Yan X Guo SB . Selective blocking of CXCR2 prevents and reverses atrial fibrillation in spontaneously hypertensive rats. J Cell Mol Med. (2020) 24:11272–82. doi: 10.1111/jcmm.15694 32812337 PMC7576251

[129] Liu P Sun H Zhou X Wang Q Gao F Fu Y . CXCL12/CXCR4 axis as a key mediator in atrial fibrillation via bioinformatics analysis and functional identification. Cell Death Dis. (2021) 12:813. doi: 10.1038/s41419-021-04109-5 34453039 PMC8397768

[130] Liehn EA Kanzler I Konschalla S Kroh A Simsekyilmaz S Sönmez TT . Compartmentalized protective and detrimental effects of endogenous macrophage migration-inhibitory factor mediated by CXCR2 in a mouse model of myocardial ischemia/reperfusion. Arterioscler Thromb Vasc Biol. (2013) 33:2180–6. doi: 10.1161/atvbaha.113.301633 23868943 PMC4337944

[131] Thiele M Kerschbaumer RJ Tam FW Völkel D Douillard P Schinagl A . Selective targeting of a disease-related conformational isoform of macrophage migration inhibitory factor ameliorates inflammatory conditions. J Immunol. (2015) 195:2343–52. doi: 10.4049/jimmunol.1500572 26209628 PMC4543907

[132] Skeens E Gadzuk-Shea M Shah D Bhandari V Schweppe DK Berlow RB . Redox-dependent structure and dynamics of macrophage migration inhibitory factor reveal sites of latent allostery. Structure. (2022) 30:840–850.e846. doi: 10.1016/j.str.2022.03.007 35381187

[133] Sajko S Skeens E SChinagl A Ferhat M Mirkina I Mayer J . Redox-dependent plasticity of oxMIF facilitates its interaction with CD74 and therapeutic antibodies. Redox Biol. (2024) 75:103264. doi: 10.1016/j.redox.2024.103264 38972295 PMC11263951

[134] Thiele M Donnelly SC Mitchell RA . OxMIF: a druggable isoform of macrophage migration inhibitory factor in cancer and inflammatory diseases. J Immunother Cancer. (2022) 10:e005475. doi: 10.1136/jitc-2022-005475 36180072 PMC9528626

[135] Mahalingam D Patel MR Sachdev JC Hart LL Halama N Ramanathan RK . Phase I study of imalumab (BAX69), a fully human recombinant antioxidized macrophage migration inhibitory factor antibody in advanced solid tumours. Br J Clin Pharmacol. (2020) 86:1836–48. doi: 10.1111/bcp.14289 32207164 PMC7444762

[136] Rossmueller G Mirkina I Maurer B Hoeld V Mayer J Thiele M . Preclinical evaluation of ON203, a novel bioengineered mAb targeting oxidized macrophage migration inhibitory factor as an anticancer therapeutic. Mol Cancer Ther. (2023) 22:555–69. doi: 10.1158/1535-7163.Mct-22-0676 37067909 PMC10157364

[137] Ferhat M Mayer J Costa LH Prendecki M Tarazona AAP Schinagl A . Targeting of oxidized macrophage migration inhibitory factor (oxMIF) with antibody ON104 attenuates the severity of glomerulonephritis. PloS One. (2024) 19:e0311837. doi: 10.1371/journal.pone.0311837 39374239 PMC11458038

[138] Ferhat M Mangano K Mirkina I Mayer J Rossmueller G Schinagl A . The newly engineered monoclonal antibody ON104, targeting the oxidized macrophage migration inhibitory factor (oxMIF), ameliorates clinical and histopathological signs of collagen-induced arthritis. Eur J Pharmacol. (2023) 956:175997. doi: 10.1016/j.ejphar.2023.175997 37579967

[139] Müller-Schiffmann A Torres F Kitaygorodskyy A Ramani A Alatza A Tschirner SK . Oxidized MIF is an Alzheimer's disease drug target relaying external risk factors to tau pathology. Cell Rep Med. (2026) 7:102520. doi: 10.1016/j.xcrm.2025.102520 41418773 PMC12866179

[140] Wei T Zhu Z Liu L Liu B Wu M Zhang W . Circulating levels of cytokines and risk of cardiovascular disease: a Mendelian randomization study. Front Immunol. (2023) 14:1175421. doi: 10.3389/fimmu.2023.1175421 37304261 PMC10247976

[141] Pu Y Zhou Y Guo T Pan X Sun X Yang G . MIF promotes phenotypic switching of VSMCs via AKT/mTOR-mediated autophagy regulation in aortic dissection. FASEB J. (2025) 39:e71079. doi: 10.1096/fj.202501761R 41001812

[142] Vámos E Kálmán N Sturm EM Nayak BB Teppan J Vántus VB . Highly selective MIF ketonase inhibitor KRP-6 diminishes M1 macrophage polarization and metabolic reprogramming. Antioxidants (Basel). (2023) 12:1790. doi: 10.3390/antiox12101790 37891870 PMC10604361

[143] Cui J Zhang F Wang Y Liu J Ming X Hou J . Macrophage migration inhibitory factor promotes cardiac stem cell proliferation and endothelial differentiation through the activation of the PI3K/Akt/mTOR and AMPK pathways. Int J Mol Med. (2016) 37:1299–309. doi: 10.3892/ijmm.2016.2542 27035848 PMC4829139

[144] Bai F Asojo OA Cirillo P Ciustea M Ledizet M Aristoff PA . A novel allosteric inhibitor of macrophage migration inhibitory factor (MIF). J Biol Chem. (2012) 287:30653–63. doi: 10.1074/jbc.M112.385583 22782901 PMC3436310

[145] Cho Y Crichlow GV Vermeire JJ Leng L Du X Hodsdon ME . Allosteric inhibition of macrophage migration inhibitory factor revealed by ibudilast. Proc Natl Acad Sci USA. (2010) 107:11313–8. doi: 10.1073/pnas.1002716107 20534506 PMC2895110

